# Impact of genetic variants in the solute carrier (*SLC*) genes encoding drug uptake transporters on the response to anticancer chemotherapy

**DOI:** 10.20517/cdr.2024.42

**Published:** 2024-07-18

**Authors:** Jose J. G. Marin, Maria A. Serrano, Elisa Herraez, Elisa Lozano, Sara Ortiz-Rivero, Laura Perez-Silva, Maria Reviejo, Oscar Briz

**Affiliations:** ^1^Experimental Hepatology and Drug Targeting (HEVEPHARM), University of Salamanca, Institute for Biomedical Research of Salamanca (IBSAL), Salamanca 37007, Spain.; ^2^Center for the Study of Liver and Gastrointestinal Diseases (CIBEREHD), Carlos III National Institute of Health, Madrid 28029, Spain.

**Keywords:** Cancer, chemotherapy, pharmacogenetics, single nucleotide alteration, single nucleotide polymorphism, transportome

## Abstract

Cancer drug resistance constitutes a severe limitation for the satisfactory outcome of these patients. This is a complex problem due to the co-existence in cancer cells of multiple and synergistic mechanisms of chemoresistance (MOC). These mechanisms are accounted for by the expression of a set of genes included in the so-called resistome, whose effectiveness often leads to a lack of response to pharmacological treatment. Additionally, genetic variants affecting these genes further increase the complexity of the question. This review focuses on a set of genes encoding members of the transportome involved in drug uptake, which have been classified into the MOC-1A subgroup of the resistome. These proteins belong to the solute carrier (SLC) superfamily. More precisely, we have considered here several members of families SLC2, SLC7, SLC19, SLC22, SLCO, SLC28, SLC29, SLC31, SLC46, and SLC47 due to the impact of their expression and genetic variants in anticancer drug uptake by tumor cells or, in some cases, general bioavailability. Changes in their expression levels and the appearance of genetic variants can contribute to the Darwinian selection of more resistant clones and, hence, to the development of a more malignant phenotype. Accordingly, to address this issue in future personalized medicine, it is necessary to characterize both changes in resistome genes that can affect their function. It is also essential to consider the time-dependent dimension of these features, as the genetic expression and the appearance of genetic variants can change during tumor progression and in response to treatment.

## INTRODUCTION

One of the main problems in cancer treatment is the poor response of many tumors to standard drug regimens. This situation can be partly explained by the existence of complex and varied mechanisms of chemoresistance (MOC), such as those leading to a reduction in the intracellular concentration of active antitumor agents^[1]^. The reduction of intracellular drug concentrations by impaired uptake markedly affects the overall effectiveness because the mechanism of action of many anticancer agents takes place inside cells, frequently inhibiting essential processes required for tumor cell survival^[1]^. Besides drug uptake, other mechanisms also contribute to the refractoriness of tumors to currently available antitumor chemotherapy, such as changes in the intracellular metabolism of antitumor drugs and prodrugs, alteration of the molecular targets, increased repair of drug-induced DNA damage, change in the balance between proapoptotic and antiapoptotic factors, modification of tumor microenvironment, and phenotypic transformations of tumor cells^[2,3]^.

Here, we have gathered the available information on the impact on cancer chemoresistance of genetic variants affecting part of the so-called “transportome,” i.e., the set of transporters expressed at a given time. The subset of transportome accounting for drug uptake belongs to the superfamily of solute carriers (SLC) proteins. Genetic variants in *SLC* genes have been associated with interindividual differences regarding drug efficacy and toxicity^[4]^. The transfer of anticancer agents across the plasma membrane of tumor cells and, hence, their effectiveness depends on the function of these proteins^[5]^. Consequently, variants affecting these genes could modify the response of tumor cells to their substrates. The relevance of some of these proteins in the transport of anticancer drugs has already been described^[6]^. Transporters encoded by the *SLC22A* gene family can accept various antitumor drugs of cationic, anionic, or zwitterionic nature as substrates. Organic anion-transporting polypeptides (OATP) encoded by *SLCO* genes transport some drugs of anionic or zwitterionic nature. In addition, certain members of the *SLC28A* and *SLC29A* gene families encode transporters capable of facilitating concentrative (CNT, concentrative nucleoside transporters) and equilibrative (ENT, equilibrative nucleoside transporters) cellular uptake of nucleoside analogs, such as gemcitabine and cytarabine^[7]^, as well as fluoropyrimidines like 5’-deoxy-5-fluorouridine^[8]^. However, they do not transport other antitumor drugs commonly used related compounds, such as 5-fluorouracil (5-FU). Moreover, the copper transporter 1 (CTR1, *SLC31A1*) mediates the cellular uptake of cisplatin. The role of other SLC transporters in the uptake of antitumor drugs is restricted to specific drugs^[5]^.

The occurrence of genetic mutations in tumor cells, driven by stochastic events, favors the selection of tumor cells that adapt to pharmacological pressure, thereby increasing tumor heterogeneity. Furthermore, in a heterogeneous population of tumor cells, Darwinian evolutionary selection can act on those showing phenotypic variation induced *de novo* as well as those carrying pre-existing variants^[9]^. Selected clones may advantageously expand, contributing to the transformation into a more chemoresistant phenotype.

In this study, we have followed the Human Genome Variation Society (HGVS) nomenclature for the description of genetic variants, according to the recommendations of the HGVS Variant Nomenclature Committee (HVNC) (https://varnomen.hgvs.org/), updated on May 1, 2020, which operates under the auspices of the Human Genome Organization (HUGO). Single nucleotide polymorphisms (SNP) have been considered substitutions in a population with a frequency higher than 1%. In contrast, single nucleotide changes without frequency limitation that can arise in tumor cells have been termed single nucleotide variations (SNV). Some of the information contained in this review has been collected from databases such as the Pharmacogene Variation Consortium (https://www.pharmvar.org), the ClinicalGenome (ClinGen) resource (https://clinicalgenome.org/), the PharmGKB database (http://www.pharmgkb.org/), the COSMIC database (https://cancer.sanger.ac.uk/cosmic), and the NCBI database of single nucleotide polymorphisms (dbSNP) (https://www.ncbi.nlm.nih.gov/snp/). The clinical significance of the various gene variants has primarily been evaluated based on the information on pharmacogenomics provided by the PharmGKB database. This database gathers studies on the phenotypic effects of the variants, assigning a score ranging from 1 to 4 based on the level of evidence, with level 1 indicating the highest criteria are met. In most cases, the clinical annotation was at level 3, which indicates a low level of evidence supporting the variant-drug association. This is because there is only one study annotated in PharmGKB, several studies have failed to replicate the association, or the annotation is based on preliminary evidence. In some cases, the level of evidence was 4, which describes variant-drug combinations where the evidence does not support a conclusive association between the variant and the phenotype.

## THE SLCO FAMILY OF ORGANIC ANION TRANSPORTING POLYPEPTIDES

OATPs constitute a superfamily of proteins that mediate the transport of amphiphilic substrates across the plasma membrane of animal cells. Although more than 300 OATP proteins have been identified, only a few have been well-characterized in humans and rodents^[10]^. Based on amino acid identity, the 11 known human OATPs have been classified into six families, containing those with more than 40% identity and ten subfamilies that encompass proteins with more than 60% identity^[11]^. OATPs carry out the sodium-independent uptake of a wide range of amphiphilic organic anions and, less frequently, neutral or cationic compounds^[11]^. They can transport a broad range of substrates with significant overlapping specificity among members of this family. These carriers are expressed in many different types of healthy cells. This expression is heterogeneously preserved in cancers derived from them. Herein lies their importance, as OATPs are involved in the uptake of some anticancer drugs, mainly negatively charged compounds^[12]^.

**Figure 1:**
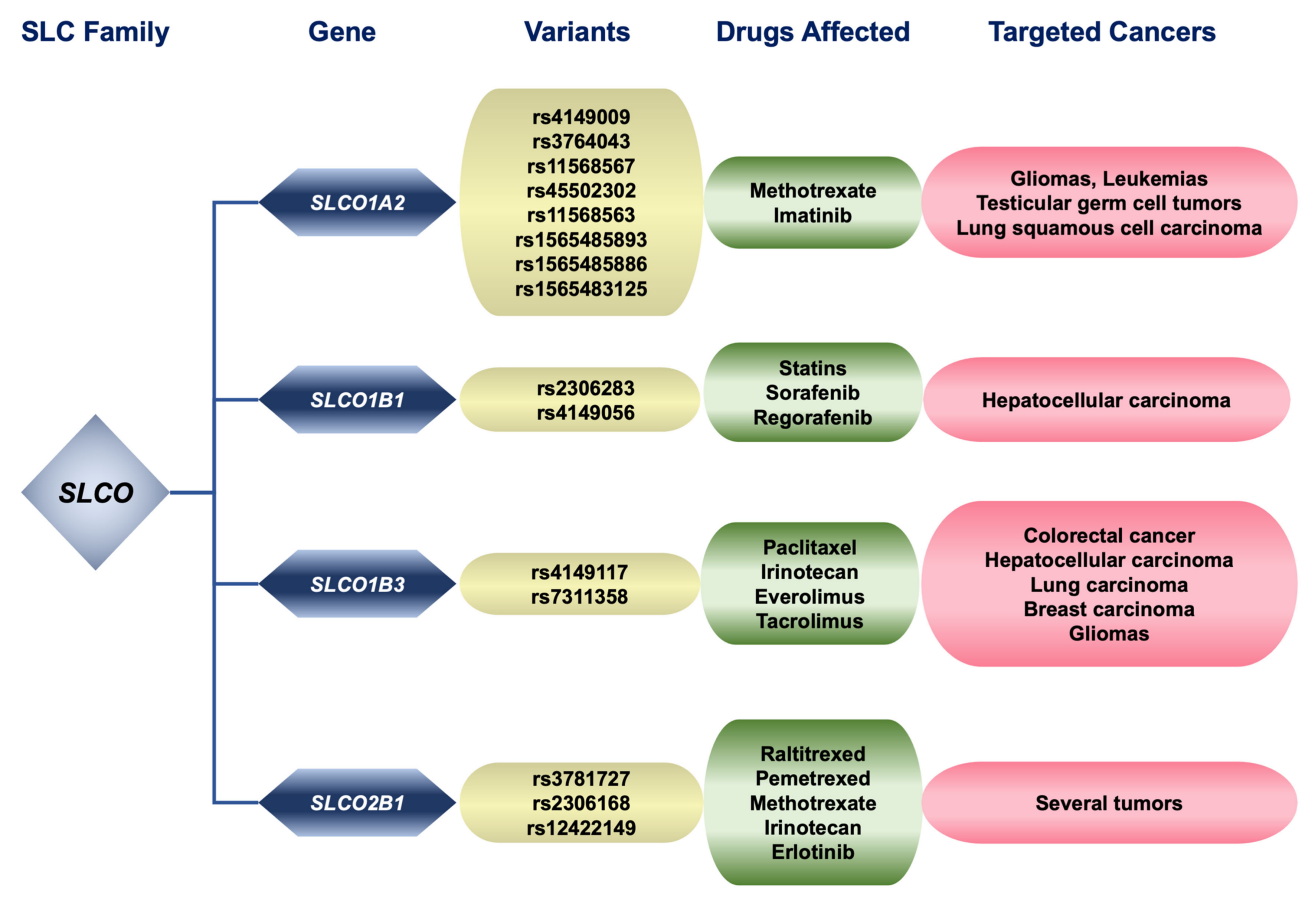
Relationship between genetic variants of pharmacologically relevant members of the SLC family SLCO, substrate drugs affected by changes in these proteins, and cancers treated with these compounds. SLC: Solute carrier.

OATP1A2 (*SLCO1A2*) is expressed mainly in the apical membrane of epithelial cells of the biliary tree, gallbladder, and digestive tract^[13]^. The highest levels of OATP1A2 expression in cancer cells are found in gliomas, testicular germ cell tumors, and squamous cell lung carcinoma^[5]^. This transporter has a broad substrate specificity that includes endogenous substrates, drugs, and other xenobiotics with anionic, neutral, or cationic nature. Regarding antitumor drugs, *in vitro* studies have reported that OATP1A2 may be involved in the uptake of methotrexate^[12,14]^ and imatinib^[15]^. Moreover, there is evidence that some *SLCO1A2* genetic variants cause a reduced or complete loss of the ability to transport these drugs. This is the case of the intronic variants g.21420471C>T (rs4149009) and g.21488004C>T (rs3764043), which could have a clinical impact on the treatment of leukemias^[16,17]^ [Figure 1 and Table 1].

**Table 1:** Clinically relevant genetic variants of members of the SLCO family of SLC in the pharmacokinetics, efficacy, or toxicity of antitumor drugs

**Variant**		**Drug**	**Targeted tumor**	**Alteration**	**Study size**	**Ethnicity**	**Ref.**
*SLCO1A2*	rs4149009	Methotrexate	Lymphoblastic leukemia	Pharmacokinetics	141	East Asian	[18]
	rs3764043	Imatinib	Chronic myeloid leukemia	Pharmacokinetics	134	East Asian	[16]
*SLCO1B1*	rs2306283	Irinotecan	Colorectal carcinoma	Efficacy	137	East Asian	[19]
		Methotrexate	Lymphoblastic leukemia	Pharmacokinetics	499	European	[20]
		Sorafenib	Hepatocellular carcinoma	Toxicity	114	European	[21]
	rs4149056	Docetaxel	Breast carcinoma	Toxicity	50	European	[22]
		Methotrexate	Lymphoblastic leukemia	Pharmacokinetics	48	Near Eastern	[23]
		Methotrexate	Lymphoblastic leukemia	Efficacy	317	East Asian	[24]
		SN-38	Non-small cell lung cancer	Pharmacokinetics	107	East Asian	[25]
		Sorafenib	Hepatocellular carcinoma	Toxicity	114	European	[21]
*SLCO1B3*	rs4149117	Paclitaxel	Non-small cell lung cancer	Toxicity	194	East Asian	[26]
		Sunitinib	Gastrointestinal stromal tumors	Efficacy	127	European	[27]
	rs7311358	Docetaxel	Nasopharyngeal neoplasms	Pharmacokinetics	50	East Asian	[28]
		Paclitaxel	Non-small cell lung cancer	Toxicity	194	East Asian	[26]

SLCO: Family of organic anion transporting polypeptides (OATPs enconded by *SLCO* genes); SLC: solute carrier.

According to the PharmGKB pharmacogenomics database, the clinical relevance of these variants is low, with a level of evidence of 3 on a scale from 1 to 4, where level 1 meets the highest criteria.

A study of 141 children with acute lymphoblastic leukemia showed that the presence of the rs4149009 variant altered the pharmacokinetics of methotrexate^[18]^. Similarly, another analysis of 34 patients with chronic myeloid leukemia and 100 controls reported that the rs3764043 variant alters the pharmacokinetics of imatinib^[16]^. Because only one clinical study has been carried out for each variant, their clinical relevance is still low. Moreover, variants affecting the ORF, such as c.382A>T (p.Asn128Tyr, rs11568567), c.404A>T (p.Asn135Ile, rs45502302), c.516A>T/C (p.Glu172Asp, rs11568563), c.550G>A (p.Glu184Lys, rs1565485893), c.553G>A (p.Asp185Asn, rs1565485886), and c.862G>A (p.Asp288Asn, rs1565483125), encode proteins that are not expressed in the plasma membrane or generate truncated inactive proteins^[29]^. However, no clinical impact of these variants has been reported, possibly because the contribution of other SLC transporters to the uptake of these drugs is higher than that of OATP1A2.

OATP1B1 (*SLCO1B1*) and OATP1B3 (*SLCO1B3*) are considered among the most clinically relevant transporters by the International Transporter Consortium (ITC) guidelines due to their role in drug uptake and disposition^[30]^. These proteins are expressed almost exclusively (OATP1B1) or abundantly (OATP1B3) in the sinusoidal membrane of hepatocytes^[31]^. As for their expression in tumors, both transporters have high levels in hepatocellular carcinoma (HCC) cells^[5]^. In addition, the expression of a splice variant of *SLCO1B3,* termed the cancer-type isoform, has been detected mainly in tumors derived from the gastrointestinal tract^[32-34]^. The clinical relevance of OATP1B1 and OATP1B3 as transport systems for antitumor agents in cancer cells is considered low due to their almost restricted localization to liver cells and limited substrate specificity for anticancer agents. Both transporters can mediate the uptake of taxanes (paclitaxel and docetaxel)^[35]^ and other antitumor drugs that are not commonly used in treating HCC^[36]^.

For this reason, the evaluation of OATP1B3 expression in HCC could be helpful before deciding whether to use anticancer drug substrates of this carrier in the personalized treatment of these patients^[36]^. When expressed in HEK293 cells *in vitro*, both OATPs can transport a wide range of tyrosine kinase inhibitors (TKIs)^[37]^. However, their relevance *in vivo* needs to be clarified because the transport of these drugs appears to be restricted to their conjugates with glucuronic acid^[38]^. OATP1B1, but not OATP1B3, has been reported to be involved in the transport of regorafenib, a TKI used in second-line treatment of advanced liver cancer, which has not responded to sorafenib^[39]^. OATP1B3 can also interact, although whether transport takes place is controversial, with mTOR inhibitors, such as everolimus^[40]^ and tacrolimus^[41]^, used against lung^[42]^ and breast carcinomas^[43]^ and gliomas^[44]^. The low expression of OATP1B3 in these tumors may be related to their low sensitivity to these drugs.

Approximately 200 variants relevant to drug transport have been described for the *SLCO1B1* gene^[45]^. Some are highly prevalent, such as c.388A>G (p.Asn130Asp, rs2306283). The c.521T>C variant (p.Val174Ala, rs4149056) leads to diminished transport activity attributable to decreased plasma membrane expression and phosphorylation status^[46]^. The most significant clinical relevance of these SNPs concerns their impact on statin uptake^[47]^. They have a moderate effect on the pharmacokinetics, drug response, and toxicity of some antitumor drugs [Figure 1 and Table 1]^[48]^. These variants have been associated with increased side effects after treatment of HCC patients with sorafenib. Still, no association with patient survival has been found in a study with 114 HCC patients^[21]^. In another study which included 499 children with acute lymphoblastic leukemia, both variants were shown to affect pharmacokinetics and response to methotrexate^[20]^.

*SLCO1B1* is one of the critical genes involved in the implementation of pharmacogenomics. Based on the studies on the impact of variants of this gene, numerous dosing recommendations based on genotype or whether a drug is indicated or contraindicated have been implemented in clinical guidelines^[49]^. However, the most critical clinical annotations on *SLCO1B1* pharmacogenetics in clinical guidelines and Food and Drug Administration (FDA)-approved drug labels all refer to statins^[45]^. Further studies with other OATP1B1 substrates, such as thiazolidinediones, antibiotics, antihypertensives, antidiabetics, and antitumor agents, are needed to reach the relevance of the statin studies.

*SLCO1B3* is also highly polymorphic, and based on the results of *in vitro* experiments, many genetic variants associated with reduced transport activity or expression have been described^[50]^. Among them, the most clinically relevant common variants are c.334T>G (p.Ser112Ala, rs4149117) and c.699G>A (p.Met233Ile, rs7311358) [Figure 1 and Table 1]^[50]^. Both have been described to lead to altered cellular localization and a reduced ability to transport taxanes so that their expression in tumor cells may contribute to interindividual variability in the pharmacokinetics of the drug and, therefore, to its antitumor activity as demonstrated in a study of 194 patients with non-small cell lung cancer treated with chemotherapy including paclitaxel^[26]^. In another study of patients with unresectable liver metastases from colorectal cancer treated with irinotecan (a substrate of OATP1B3) and other OATP1B3 non-transported drugs (oxaliplatin and 5-FU), the presence of these variants has been linked to altered pharmacokinetics of irinotecan, resulting in reduced hepatic detoxification and consequently increasing its adverse effects, such as neutropenia and diarrhea^[51]^. More studies are needed for the level of evidence of an association between *SLCO1B3* variant-drug combinations to be considered higher.

Recently, the ITC guidelines have included OATP2B1 (*SLCO2B1*) among the transporters of emerging clinical relevance due to its essential role in drug absorption and disposition^[30]^. OATP2B1 shows widespread tissue expression, being particularly abundant in the liver and intestine^[52]^. Recent proteomic data indicate that OATP2B1 has a similar expression to OATP1B3 in the liver^[53]^, suggesting that its importance in hepatic uptake may have been underestimated. Its expression in tumors is relatively high in most types of cancer, except in acute myeloid leukemia (AML)^[5]^. Although OATP2B1 has a broad substrate specificity among anionic organic compounds, both endogenous and xenobiotics^[31,54]^, few of its substrates are antitumor drugs. OATP2B1 has been demonstrated to play a role in the intestinal absorption of antifolate drugs such as raltitrexed, pemetrexed, and methotrexate^[55]^, as well as the most active metabolite of irinotecan, SN-38^[56]^.

Numerous TKIs are inhibitors of OATP2B1, most notably erlotinib, but whether they are substrates of this transporter is unknown^[57]^. Results from *in vitro* experiments have shown that several *SLCO2B1* variants affect protein expression or function, such as g. 75204976T>C (rs3781727), c.1457C>T (p.Ser486Phe, rs2306168), c.935G>A/T (p.Arg312Gln/Leu, rs12422149), but its clinical relevance for antitumor drugs has not yet been found^[58]^.

Although other members of the OATP family are expressed in some types of tumors and can transport antitumor drugs, such as OATP1C1 (*SLCO1C1*), which transports docetaxel^[59]^, OATP4C1 (*SLCO4C1*), methotrexate^[60]^, and OATP5A1 (*SLCO5A1*), satraplatin^[61]^, their role in cancer chemoresistance is poorly understood.

## THE SLC22 FAMILY

Within the SLC superfamily of transporter proteins, the SLC22 family plays critical roles in physiology, pharmacology, and toxicology due to its ability to transport a wide variety of substrates and the expression of some of its members in crucial organs involved in drug disposition, such as the intestine, liver, and kidney^[62]^. Not all twenty-four transporters of the SLC22 family have been well characterized^[63]^. Phylogenetic studies have classified them into six subfamilies, of which the three best known are those of organic cation transporters (OCT), organic cation/carnitine transporters (OCTN), and organic anion transporters (OAT), in addition to others less well-known, such as OAT-like, OAT-related, and OCT/OCTN-related transporters^[63,64]^. A common feature of all SLC22 proteins is their structure, which consists of 12 transmembrane domains (TMD) with three highly conserved areas that are important for their function: a large extracellular loop at the beginning, between TMD1 and TMD2; another large intracellular loop in the central region, between TMD6 and TMD7; and motifs in TMD9 and TMD10 that are crucial for the transport activity of the protein^[65]^. Current U.S. FDA and European Medicines Agency (EMA) guidelines consider OCT transporters as proteins of great importance in pharmacology^[48,63]^.

### OCTs

OCT1 (*SLC22A1*) is primarily a hepatic uptake transporter expressed in the sinusoidal membrane of hepatocytes, where it mediates the uptake from portal blood of a wide variety of endogenous compounds and cationic drugs^[66]^. OCT1 promiscuously transports structurally diverse endogenous organic cations, such as thiamine, choline, and cationic neurotransmitters, and many commonly used drugs, such as metformin, ranitidine, sumatriptan, or lamivudine^[64]^. Among the antitumor drugs, irinotecan, mitoxantrone, oxaliplatin, paclitaxel, imatinib, and sorafenib have been described as OCT1 substrates^[67,68]^.

**Figure 2:**
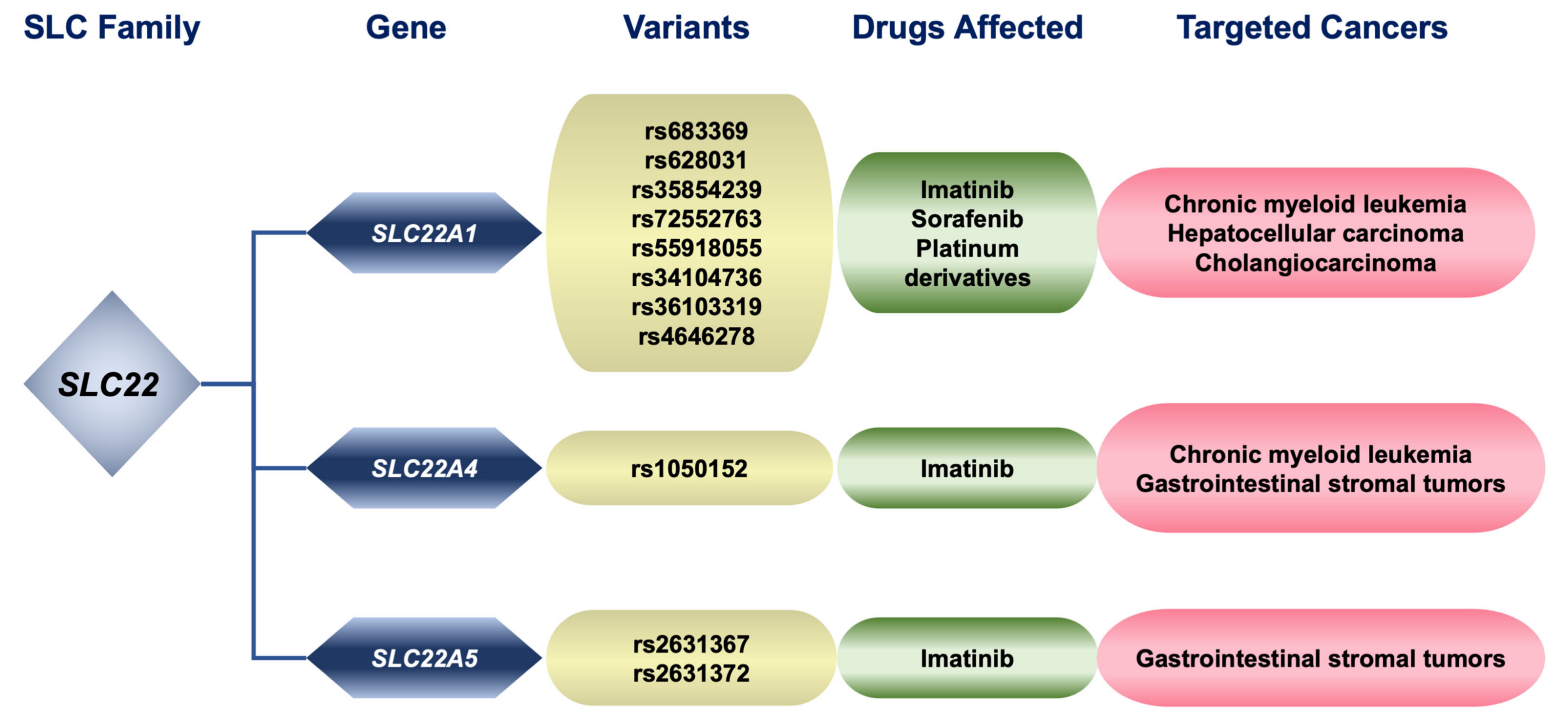
Relationship between genetic variants of pharmacologically relevant members of the SLC family 22, substrate drugs affected by changes in these proteins, and cancers treated with these compounds. SLC: Solute carrier; SLCO: family of organic anion transporting polypeptides (OATPs enconded by *SLCO* genes).

The results obtained from *in vitro* and *in vivo* studies, as well as in numerous clinical investigations, support the functional impact of some genetic variants of OCT1 on the pharmacokinetics and chemotherapeutic response of drugs that are substrates of this transporter [Figure 2 and Table 2].

**Table 2:** Clinically relevant genetic variants of members of the SLC22A family of SLC in the pharmacokinetics, efficacy, or toxicity of antitumor drugs

**Variant**		**Drug**	**Targeted tumor**	**Alteration**	**Study size**	**Ethnicity**	**Ref.**
*SLC22A1*	rs683369	Imatinib	Chronic myeloid leukemia	Efficacy	60	European	[69]
		Imatinib	Gastrointestinal stromal tumors	Toxicity	118	East Asian	[70]
	rs628031	Imatinib	Chronic myeloid leukemia	Efficacy	642	Multiple groups	[71]
		Imatinib	Chronic myeloid leukemia	Pharmacokinetics	38	East Asian	[72]
		Imatinib	Gastrointestinal stromal tumors	Toxicity	38	European	[73]
*SLC22A4*	rs1050152	Imatinib	Chronic myeloid leukemia	Efficacy	189	Multiple groups	[74]
		Imatinib	Gastrointestinal stromal tumors	Efficacy	54	European	[75]
*SLC22A5*	rs2631367	Imatinib	Gastrointestinal stromal tumors	Efficacy	54	European	[75]
	rs2631372	Imatinib	Gastrointestinal stromal tumors	Efficacy	54	European	[75]

SLC: Solute carrier.

The information from the PharmGKB pharmacogenomics database, indicate that the clinical relevance of these variants is placed third in the range from 1 to 4.

Global genetic analyses have shown marked inter-ethnic variability regarding *SLC22A1* variants affecting OCT1 activity^[76]^. Imatinib, whose uptake has been shown to depend on OCT1^[77]^, is a potent inhibitor of the BCR-ABL tyrosine kinase and has therefore been used in the treatment of chronic myeloid leukemia (CML), where this aberrant fusion protein is very frequent (> 95% of cases). Thus, OCT1 expression and activity may be a critical determinant of intracellular drug levels^[78]^; indeed, the OCT1 expression level has been suggested to be a valuable biomarker for predicting the success of imatinib-based therapy in CML patients^[79,80]^. A meta-analysis found a significant association between the presence of the *SLC22A1* variant c.480G>A/C/T (p.Leu160Phe, rs683369) and a lower response rate to imatinib in CML patients^[71]^. Other authors reported in a study with 278 Asian CML patients that the presence of the SNP c.1222A>C/G (p.Met408Val, rs628031) together with the intron variant g.Chr6:160139868insGTAAGTTG (rs35854239), an 8-bp insertion variant that duplicates the splicing motif 8 bp downstream of the original splicing motif, and the SNP c.1260_1262del (p.Met420del, rs72552763) increase the probability of developing resistance to imatinib^[81]^. Consistent with this information, another study with 167 CML patients in the chronic phase revealed that patients with the rs628031 variant, both in homozygosis and heterozygosis, and additional rare genotypes had worse event-free survival and overall survival (OS) compared to patients with only the rs628031 variant^[82]^. The relevance of these results is limited because they could not be replicated in other trials. For example, the presence of the SNP rs628031 was only significantly correlated with imatinib pharmacokinetics when it was part of a haplotype that included rs3798168 and rs628031^[72]^.

On the other hand, in liver tumors, both in HCC and cholangiocarcinoma (CCA), many *SLC22A1* genetic variants have been found, both SNVs and aberrant splicing forms, which result in reduced or even abolished transporter function, when truncated proteins are generated^[83]^. For instance, in a high proportion (40%) of HCC analyzed, at least one aberrant splicing variant (often exon 10 skipping) has been reported^[83]^. Other examples of inactivating variants found in these tumors are c.262T>C/A (p.Cys88Ser/Arg, rs55918055), c.566C>T (p.Ser189Leu, rs34104736), c.659G>C/T (p.Gly220Ala/Val, rs36103319), and c.859C>G/T (p.Arg287Gly/Trp, rs4646278). These SNVs and others, such as c.262delT (p.Cys88Alafs*16) and c.181delCGinsT (p.Arg61Serfs*10), result in lower uptake of sorafenib (an oral multikinase inhibitor used for the treatment of HCC) and, therefore, lower induced cytotoxicity^[83]^. Similarly, the presence of OCT1 in the plasma membrane of tumor cells has been associated with a better outcome in HCC patients treated with sorafenib^[84]^. Similar variants have also been described in CCA. Although sorafenib is not currently included in the standard treatment of CCA, *in vitro* studies have demonstrated that the uptake of other drugs, such as platinum derivatives used in first-line pharmacological regimes, is affected by the appearance of inactivating variants of OCT1^[85]^.

Most studies on the clinical implications of *SLC22A1* variants have been focused on the antidiabetic drug metformin and the TKI imatinib^[67-69,71,82,86]^. However, it is essential to note the need to replicate the results obtained. Therefore, the clinical utility of these variants in cancer chemotherapy should be approached with caution at present. Larger sample sizes are needed to validate the role of *SLC22A1* variants in anticancer drug disposition, response, and toxicity.

OCT2 (*SLC22A2*) is mainly expressed in the kidney, located at the basolateral plasma membrane of proximal tubule cells^[66]^. It works with multidrug and toxin extrusion proteins MATE1 (*SLC47A1*) and MATE2 (*SLC47A2*) to eliminate many cationic and zwitterionic endogenous compounds and drugs in the urine^[87]^. Metformin and platinum derivatives are among the most relevant clinical substrates^[66]^.

More than fourteen genetic variants have been found in the *SLC22A2* gene^[88]^. However, the impact of these variants on chemotherapy response may be negligible because renal carcinoma expresses low OCT2 levels compared to normal kidney tissue^[89]^, and the antitumor drugs described as OCT2-substrates (ifosfamide and platinum derivatives)^[85,90]^ are not used in the treatment of renal carcinoma.

*SLC22A3* missense variants are rare throughout all populations, and none of all OCT3 variants described so far result in a complete loss of function, nor have they been associated with changes in pharmacokinetics or pharmacodynamics^[91]^. Only the SNVs c.1110G>T (p.Met370Ile, rs137958808) and c.1199C>A/T (p.Thr400Asn/Ile, rs8187725) have functional consequences *in vitro* and show a partially reduced uptake of well-characterized substrates^[91]^. Although there is no described connection between *SLC22A3* variants and response to chemotherapy in patients, it has been reported that some genetic variants of *SLC22A3* in the 5’-flanking region may modulate OCT3 expression^[92]^.

### OCTNs

OCTN1 (*SLC22A4*) and OCTN2 (*SLC22A5*) are transporters of carnitine, acetylcholine, and ergothioneine widely distributed in the organism. OCTN1 is abundantly expressed in the digestive tract and biliary system, as well as in the bone marrow, prostate, lung, and kidney. In contrast, OCTN2 is mainly expressed in the kidney, intestine, and prostate^[93]^. Regarding antitumor drugs, OCTN1 participates in the uptake of mitoxantrone^[94]^, doxorubicin^[94]^, oxaliplatin^[95]^, imatinib^[15]^, and nucleoside derivatives, such as cytarabine, 2’-deoxycytidine, and gemcitabine^[96]^. On the other hand, the substrate specificity concerning antitumor drugs for OCTN2 is more restricted, including, for instance, etoposide^[97]^ and imatinib^[15]^.

A relationship between some OCTN1/2 polymorphisms and the prognosis of some gastrointestinal tumors treated with imatinib has been established^[75]^ [Figure 2]. In this line, in patients with gastrointestinal stromal tumors treated with imatinib, the time to progression period was significantly improved in carriers of the C allele of the *SLC22A4* variant c.1507C>T (p.Leu503Phe, rs1050152), as well as in carriers of the minor alleles of the *SLC22A5* variants c.-207C>G/A/T (rs2631367) and c.-2087G>C (rs2631372), both located at the promoter, suggesting that OCTN1 and OCTN2 activity or expression may predict the efficacy of imatinib chemotherapy^[98]^. Some *SLC22A4* variants can modulate imatinib response in patients with CML, where this drug is also used. For instance, an association has been found between the SNP rs1050152 of OCTN1 and the response to imatinib in CML patients^[74]^.

### OATs

These proteins are mainly expressed in the kidneys and liver and at lower levels, in the brain, placenta, prostate, and testis^[99]^. Regarding their activity, OATs can transport small and negatively charged endogenous compounds, including metabolites, signaling molecules, nutrients, gut microbiome products, antioxidants, and uremic toxins. Among their substrates are antivirals, antibiotics, nonsteroidal anti-inflammatory drugs, antihypertensives, diuretics, and many other clinically relevant drugs (for a review^[100]^). However, OATs have a scarce impact on antitumor drug uptake. In this line, although different genetic and splice variants have been described, no information is available on the effect of these variants in antitumor drug response.

Methotrexate, used in the treatment of several cancers (uterus, breast, and lung carcinomas, certain cancers of the head and neck, and some lymphomas and leukemias), has been described as a substrate of OAT1 (*SLC22A6*)^[101]^ and OAT2 (*SLC22A7*)^[102]^. Moreover, OAT2 can participate in the uptake of irinotecan^[103]^ and probably 5-FU^[104]^, which is consistent with the fact that the high expression of this transporter has been proposed as a predictor of effectiveness for 5-FU-based chemotherapeutic regimens such as FOLFOX (5-FU/leucovorin/oxaliplatin) in metastatic colorectal cancer^[105]^. Several genetic polymorphisms have been described in the *SLC22A7* gene^[106]^, and a splice variant containing an additional nucleotide sequence of the intron 1 (TCCCAG) between exons 1 and 2 of the open reading frame has been detected in liver, kidney, and pancreas in approximately the same proportion of expression as the wild-type transporter. The peptide encoded by this variant is not functional because it is retained intracellularly and consequently lacks transport activity^[107]^. However, the impact of these OAT2 genetic variants on drug response has yet to be elucidated.

## THE SLC28A AND SLC29A FAMILIES OF NUCLEOSIDE TRANSPORTERS

The *SLC28* and *SLC29* gene families encode transporters accounting for the uptake of natural nitrogen bases, nucleosides, and nucleotides. They are the main pathways to crossing the plasma membrane for many purine and pyrimidine analogs used in anticancer therapies for various tumors^[108-111]^.

### CNTs

**Figure 3:**
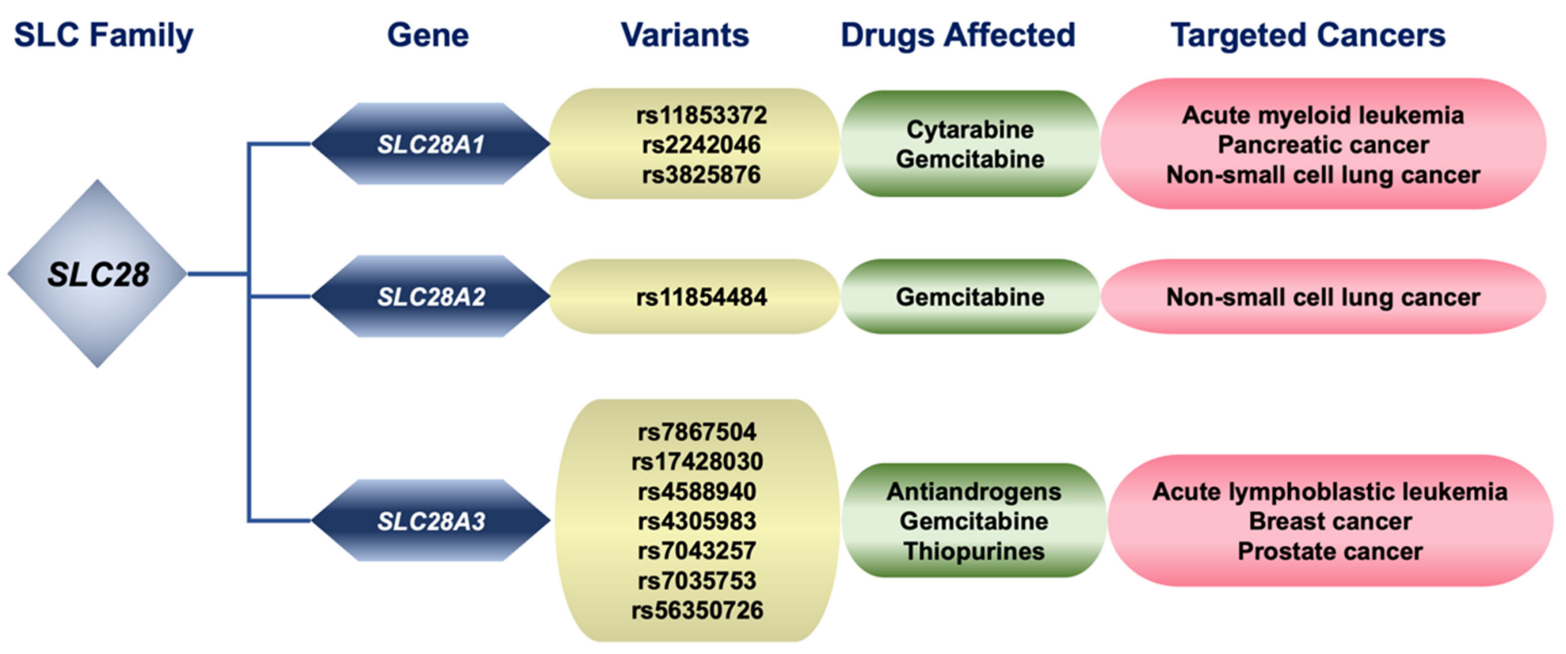
Relationship between genetic variants of pharmacologically relevant members of the SLC family 28, substrate drugs affected by changes in these proteins, and cancers treated with these compounds. SLC: Solute carrier.

CNTs include three members of Na^+^-dependent secondary active transporters. CNT1 (*SLC28A1*) is predominantly found in the apical membrane of epithelial cells in the small intestine, liver, and kidney^[112]^. It mediates the uptake of pyrimidine nucleosides, including fluoropyrimidines such as gemcitabine^[113]^, and hypomethylating nucleoside analogs with antitumor activity, such as cytarabine, azacytidine, decitabine, and zebularine^[114-117]^. In addition to the loss of CNT1 expression identified in solid tumors and leukemias, where nucleoside analogs are used as first-line treatment agents^[115,118,119]^, several genetic variants have been associated with a worse outcome in patients with different types of cancer [Figure 3 and Table 3].

**Table 3:** Clinically relevant genetic variants of members of the SLC28A and SLC29A families of SLC in the pharmacokinetics, efficacy, or toxicity of antitumor drugs

**Variant**		**Drug**	**Targeted tumor**	**Alteration**	**Study size**	**Ethnicity**	**Ref.**
*SLC28A1*	rs2242046	Gemcitabine	Non-small cell lung cancer	Toxicity	53	East Asian	[120]
	rs3825876	Gemcitabine	Pancreatic cancer	Toxicity	294	European	[121]
*SLC28A2*	rs11854484	Gemcitabine	Non-small cell lung cancer	Efficacy	53	East Asian	[120]
*SLC28A3*	rs7867504	Gemcitabine	Solid tumors	Pharmacokinetics	40	American	[122]
*SLC29A1*	rs9394992	Tipiracil, trifluridine	Colorectal carcinoma	Efficacy	179	Multiple groups	[123]
		Gemcitabine	Pancreatic cancer	Toxicity	149	Multiple groups	[124]
	rs747199	Gemcitabine	Breast carcinoma	Efficacy	85	East Asian	[125]
	rs760370	Gemcitabine	Breast carcinoma	Efficacy	85	East Asian	[125]
		Gemcitabine	Pancreatic cancer	Efficacy	149	Multiple groups	[124]
		Tipiracil, trifluridine	Colorectal carcinoma	Efficacy	179	Multiple groups	[123]
*SLC29A3*	rs780668	Gemcitabine	Non-small cell lung cancer	Efficacy	88	Multiple groups	[126]

SLC: Solute carrier.

According to the PharmGKB pharmacogenomics database, the clinical relevance of these variants is low, with a level of evidence of 3, or 4 in the case of *SLC29A3*, on a scale from 1 to 4, where level 1 meets the highest criteria.

Thus, the *SLC28A1* intronic variant c.795+4320T>A (rs11853372) has been associated with low-intracellular cytarabine levels in cancer cells collected from children with leukemia^[127]^. A study with a heterogeneous cohort of patients with solid tumors treated with gemcitabine revealed that the rs11853372 variant was associated with impaired clearance of 2’,2’-difluoro-2’-deoxycytidine triphosphate and hence could influence gemcitabine response^[128]^. However, these studies are not considered sufficient evidence for clinical relevance by international pharmacogenomics consortia. However, there are two variants that have a moderate clinical impact. In patients with non-small cell lung cancer (NSCLC) treated with gemcitabine-based therapy, the non-synonymous *SLC28A1* variant c.1561G>A/T (p.Asp521Asn/Tyr, rs2242046) has been associated with increased myelotoxicity due to an increased gemcitabine absorption^[120]^. Another intron variant, c.277+2103G>A (rs3825876), has demonstrated a higher risk of neutropenia in advanced pancreatic cancer patients treated with this drug^[121]^.

In contrast to CNT1, CNT2 (*SLC28A2*), whose expression is high in the intestinal tract, biliary system, and kidney, prefers purine nucleosides as substrates. A limited impact of CNT2 on tumor sensitivity to drugs transported by this transporter is expected due to its low expression in most tumor types^[5]^. Nevertheless, in some cancers, for instance, gastric, colorectal, endometrial, or lung adenocarcinomas, it has been shown that impaired expression or activity of CNT2 can affect the activity of the antitumor nucleoside analogs^[129]^. A study conducted on Asian populations regarding the association of gemcitabine pharmacology with the prognosis of patients with NSCLC has suggested that the *SLC28A2* variant c.65C>A/G/T (p.Pro22Gln/Arg/Leu, rs11854484) is associated with better outcomes. However, the reason for this relationship has yet to be elucidated^[120]^. Furthermore, an SNP in the promoter region of *SLC28A2* (c.-146T>A; rs2413775), which induces increased transcription of this gene, has been suggested to play a role in the interindividual variability of pharmacokinetics and pharmacological effects of nucleoside analogs^[130]^.

Another member of this group of transporters is CNT3 (*SLC28A3*), which has a broad substrate selectivity. Indeed, it does not discriminate between purine and pyrimidine nucleosides and can also transport various drugs with antitumor activity, such as gemcitabine, floxuridine, and zebularine^[131]^. Its expression, which is elevated in carcinomas derived from the gastrointestinal tract, pancreas, ovaries, and cervix, squamous cell carcinoma of the lung, mesothelioma, and testicular tumors, is considered a valuable predictive biomarker for the response of leukemias to chemotherapy based on cytarabine^[132]^ or thiopurines^[133]^. Regarding genetic modifications, several *SLC28A3* variants have been associated with resistance to antitumor drugs [Figure 3]. The variant c.267A>T/G/C (p.Thr89Thr, rs7867504) was associated with clinical outcomes in patients with metastatic breast cancer receiving gemcitabine plus paclitaxel chemotherapy^[125]^. Moreover, several intronic SNPs: c.60+1868T>C (rs17428030), c.61-13251T>G (rs4588940), c.60+11038T>C (rs4305983), c.60+1815A>G (rs7043257), c.-45-89G>C (rs7035753), may reduce response to thiopurines of childhood acute lymphoblastic leukemia (ALL)^[134]^. A study carried out on a Korean population with metastatic prostate cancer has found that the variant c.1538A>T/G (p.Tyr513Phe/Cys, rs56350726) was associated with metastasis and progression of castration-resistant prostate cancers, probably due to a less efficient transport androgen-deprivation therapy^[135]^.

### ENTs

**Figure 4:**
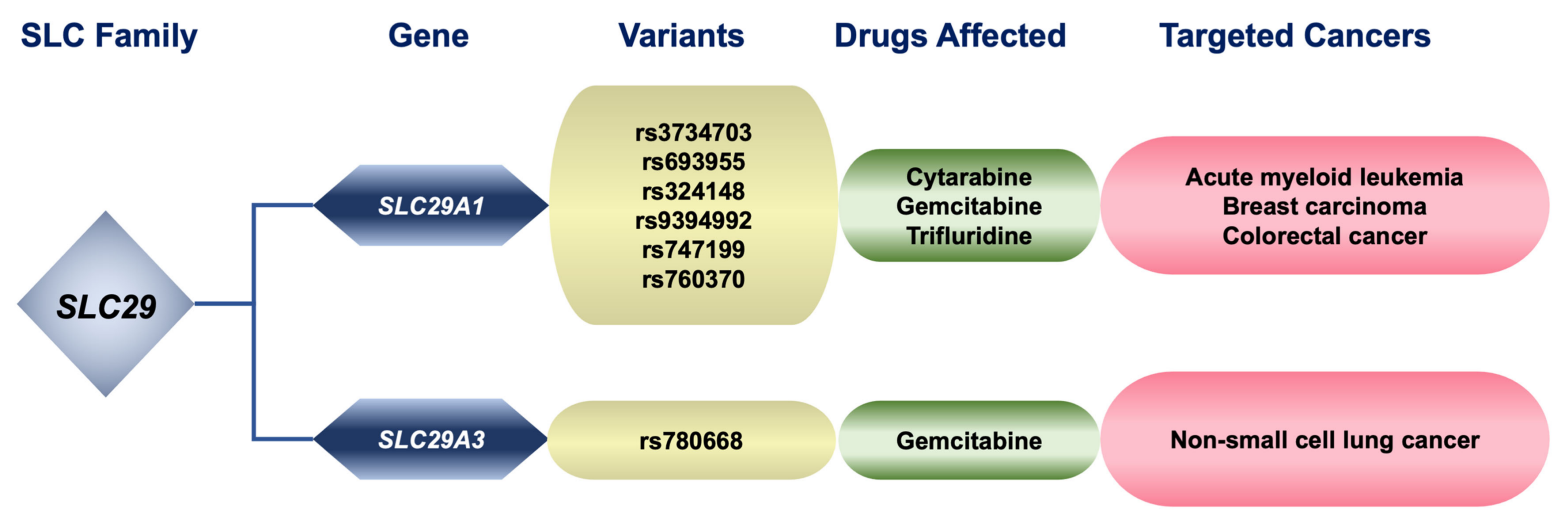
Relationship between genetic variants of pharmacologically relevant members of the SLC family 29, substrate drugs affected by changes in these proteins, and cancers treated with these compounds. SLC: Solute carrier.

The *SLC29* gene family encodes four different ENT proteins. ENT1 (*SLC29A1*) is highly expressed in most types of cancers^[5]^, where it carries out most of the facilitative uptake by tumor cells of natural nucleosides and nucleoside-derived drugs, such as gemcitabine or cytarabine^[136,137]^. Several studies have suggested potential contributions of *SLC29A1* genetic variants [Figure 4 and Table 3]: c.946-207893C>A (rs3734703), c.-162+228A>C (rs693955), c.30-549T>C (rs324148), and c.29+913C>T (rs9394992) to the cytarabine resistance and clinical outcomes in patients with AML receiving cytarabine^[138-140]^. Moreover, c.-55+441G>A (rs747199) has been associated with a poorer response of breast carcinomas to gemcitabine and paclitaxel^[125]^. Low ENT1 expression is related to the presence of the intronic variant c.1260-201A>C (rs760370), which has been suggested as a prognostic marker in patients with colorectal cancer treated with trifluridine^[141]^.

Although ENT2 (*SLC29A2*) and ENT1 are thought to play an essential role in the uptake of antitumor purine and pyrimidine analogs, ENT2 has a more restricted tissue expression profile. High *SLC29A2* mRNA levels are found in the digestive tract and tumors derived from these tissues^[5]^.

Although the genetic variability of ENT3 (*SLC29A3*), which has a high affinity for adenosine, is very low compared to ENT1, and hence, variations in its coding sequence are expected to have a low impact on the clinical response to drugs taken up by this transporter, the expression of the *SLC29A3* variant c.473C>T (p.Ser158Phe, rs780668) seems to correlate with the outcome of NSCL patients treated with gemcitabine^[126]^ [Figure 4].

## SLC31 FAMILY OF COPPER TRANSPORTERS

Proteins encoded by the *SLC31* genes, together with ATP7A and ATP7B P-type ATPases, regulate cellular copper levels. CTR1 (*SLC31A1*), expressed at the plasma membrane, is involved in the uptake of monovalent copper by the cells, whereas CTR2 (*SLC31A2*), located in intracellular membranes, is involved in vacuolar accumulation^[142]^. CTR1, which has higher substrate affinity than CTR2, is ubiquitously expressed in the body, with the highest levels found in the liver, gastrointestinal tract, kidney, and choroid plexus. Although CTR1 is expressed in many types of cancers, the highest levels are found in tumors derived from tissues where CTR1 expression is high^[5]^. In addition to copper, CTR1 can also transport other metals such as cadmium, silver, zinc, and cobalt^[142]^. Its interest in cancer pharmacology is based on the ability of this transporter to mediate the uptake of platinum-derived antitumor drugs such as cisplatin, oxaliplatin, and carboplatin^[143,144]^. Accordingly, CTR1 expression is clinically more relevant in tumors whose treatments include platinum derivatives, which is the case of reproductive, respiratory, and most gastrointestinal cancers^[5,145]^.

**Figure 5:**
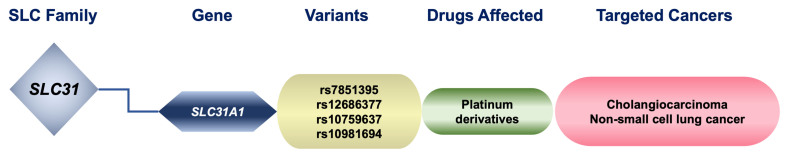
Relationship between genetic variants of pharmacologically relevant members of the SLC family 31, substrate drugs affected by changes in these proteins, and cancers treated with these compounds. SLC: Solute carrier.

Few genetic variants of *SLC31A1* have been described in healthy tissues, and there is no evidence supporting any impact of these altered proteins on the pharmacokinetics, response, and toxicity of the drugs transported by CTR1^[146]^. According to the COSMIC database of mutations in cancer, *SLC31A1* is mutated in less than 1% of samples, reaching 2.1% in HCC, 2.0% in CCA, and 1.8% in endometrial carcinoma^[147]^. Most studies on the relevance of *SLC31A1* variants in cancer chemoresistance have been carried out in patients with NSCLC, a type of cancer treated with platinum derivatives [Figure 5 and Table 4].

**Table 4:** Clinically relevant genetic variants of members of other families of SLC in the pharmacokinetics, efficacy, or toxicity of antitumor drugs

**Variant**		**Drug**	**Targeted tumor**	**Alteration**	**Study size**	**Ethnicity**	**Ref.**
*SLC31A1*	rs7851395	Carboplatin, cisplatin	Non-small cell lung cancer	Efficacy	282	East Asian	[148]
	rs10759637	Platinum compounds	Non-small cell lung cancer	Efficacy	1004	East Asian	[149]
	rs10981694	Cisplatin	Non-small cell lung cancer	Toxicity	204	East Asian	[150]
		Cisplatin	Testicular neoplasms	Toxicity	196	American	[151]
*SLC7A5*	rs4240803	Melphalan	Myeloma	Toxicity	135	American	[152]
*SLC19A1*	rs1051266	Methotrexate	Lymphoblastic leukemia	Efficacy	31	Latino	[153]
		Methotrexate	Osteosarcoma	Efficacy	62	European	[154]
		Methotrexate	Lymphoblastic leukemia	Toxicity	95	East Asian	[155]
		Methotrexate	Osteosarcoma	Toxicity	37	East Asian	[156]

SLC: Solute carrier.

In the scale of the PharmGKB pharmacogenomics database, the clinical relevance of these variants is for 3 *SLC31A1* and *SLC7A5*, and 4 in the case of *SLC19A1*.

Although intron variants are not expected to alter the protein structure (unless they favor aberrant splicing), they can markedly affect the expression levels. Thus, g.116002464A>G (rs7851395) and g.116004033C>A (rs12686377) cause CTR1 downregulation^[148]^, which has been associated with a worse response of NSCLC patients to platinum derivative therapy^[148]^. A similar association has been reported for the germinal SNP g.116025024A>C (rs10759637), which affects the 3’-UTR region of *SLC31A1* mRNA and reduces the expression of the transporter. Following intravenous administration of the platinum drug, the presence of the rs10759637 variant results in decreased platinum uptake in tissues and its accumulation in bone marrow and peripheral blood, and is therefore associated with increased platinum resistance in the tumor and the occurrence of hematological toxicity such as thrombocytopenia^[149]^. The intron variant g.113224129T>G (rs10981694) has also been associated with cisplatin-induced ototoxicity in patients with NSCLC, and it has been suggested that detection of its presence before treatment could be considered when choosing cisplatin treatment in these patients^[150]^. CCA is another cancer treated with drug combinations that include cisplatin. Although no association has been found between the presence of the SNP rs12686377 of *SLC31A1* and patient response, when this SNP appears with *ERCC1* variants, it could predict a lack of response to treatment with gemcitabine plus cisplatin^[157]^.

## THE SLC47 FAMILY OF MULTIDRUG AND TOXIN EXTRUSION TRANSPORTERS

MATE1 and MATE2 are bidirectional H^+^/organic cation transporters^[158,159]^. MATE1 was identified in 2005 as a mammalian ortholog of the MATE bacterial family that confers resistance to multiple drugs^[158]^. MATE1 is highly expressed in the liver and kidney in humans and localizes to the canalicular membrane of hepatocytes and the apical membrane of proximal tubular cells. Additionally, MATE1 is also expressed in skeletal muscle and other tissues^[160]^. MATE2 pre-mRNA can generate two major alternative splicing variants. The long one (MATE2-B) is a non-functional variant ubiquitously expressed in all tissues except the kidney. The short variant (MATE2-K) is translated into an active transporter with kidney-specific expression^[161]^. Functionally, MATE1 and MATE2-K work together as a detoxification system by mediating renal tubular secretion of intracellular ionic compounds across the brush border membranes^[158]^.

**Figure 6:**
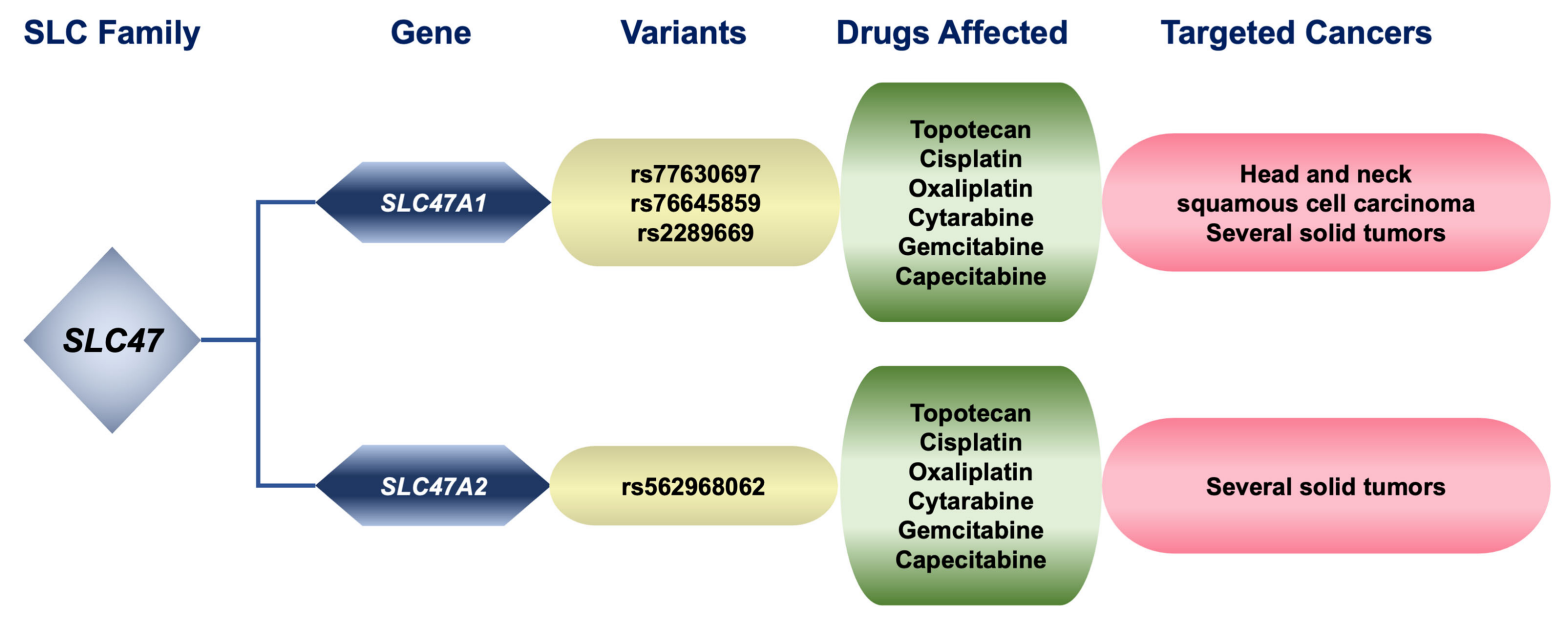
Relationship between genetic variants of pharmacologically relevant members of the SLC family 47, substrate drugs affected by changes in these proteins, and cancers treated with these compounds. SLC: Solute carrier.

The endogenous substrates of MATE1 and MATE2 include the organic cations creatinine, guanidine, and thiamine, organic anions, such as estrone sulfate, and neutral steroids, such as corticosterone^[158,159,161]^. In addition, it has been shown that around 30 drugs in clinical use were identified as MATE substrates. These include antineoplastic drugs such as topotecan^[159]^, cisplatin, oxaliplatin^[162,163]^, cytarabine, gemcitabine, and capecitabine^[164]^. More information is needed on the impact of MATE expression and impaired functions due to genetic variants in drug handling by tumor cells because, up to now, most studies have been focused on their role in the pharmacokinetics of anticancer agents. More than 16,000 SNPs and SNVs are currently listed for *SLC47A* genes in the NCBI-SNP database, most located in non-coding regions and just over 2,200 in exons. Although most studies of MATE1 and MATE2 pharmacogenetics have been performed for metformin^[86]^, a similar impact could be extrapolated to anticancer drugs transported by these proteins [Figure 6]. In a study with a cohort of patients with kidney damage, two *SLC47A1* variants have been found: c.191G>A (Gly64Asp, rs77630697) and c.1438G>A (p.Val480Met, rs76645859) and one in *SLC47A2*: c.740G>T (p.Gly211Val, rs562968062), which cause a loss of protein function. Increased accumulation of oxaliplatin has been found in the kidney, with subsequent nephrotoxicity suggesting that it could be caused by loss of function^[165]^. One study has linked the presence of *SLC47A1* variants to cisplatin-induced toxicity. In an adult cohort of patients with head and neck squamous cell carcinoma who received cisplatin treatment, the presence, in homozygosis or heterozygosis, of the *SLC47A1* variant g.19560030G>A (rs2289669) was found to predispose to cisplatin-induced toxicity^[166]^.

## OTHER SLC TRANSPORTERS

### Facilitative glucose transporters

The first four members of the SLC2A subfamily are facilitative transporters of hexoses, mainly glucose. Facilitative glucose transporter 1 (GLUT1, gene *SLC2A1*) is the major glucose transporter in the brain, placenta, and erythrocytes^[167]^. GLUT2 (gene *SLC2A2*) is a low-affinity, high-capacity transporter, which, besides being involved in glucose transport by the liver and kidney, acts as a glucose sensor by mediating glucose uptake by beta pancreas cells^[167]^. GLUT3 (*SLC2A3*) is involved in glucose transport by the placenta and brain, while GLUT4 (*SLC2A4*) is the insulin-sensitive transporter expressed in skeletal muscle, heart, and adipose tissue^[167]^. Enhanced expression of GLUTs has been observed in several types of cancer^[168]^. For instance, GLUT1 upregulation has been reported in HCC^[169]^, pancreatic tumors^[170,171]^, cervical squamous cell carcinoma^[172]^, prostate cancer, and several other cancers^[168]^. This is clinically relevant because glucose uptake is required for cancer development, progression, and metastasis. Regarding chemoresistance, evidence suggests a link between glycolysis and DNA repair mechanisms, as the glycolytic pathway provides metabolites that play an essential role in DNA metabolism^[173]^. Accordingly, glucose transporter inhibitors can be used in cancer therapy to enhance the cytostatic effect of DNA-damaging drugs like cisplatin^[168]^. The presence of *SLC2A1* genetic variants, such as g.43426591C>T (rs3738514), g.43392250C>G/T (rs4658), and g.43387302C>G/T (rs841844), has been associated with the overall toxicity of platinum-based chemotherapy in lung cancer patients^[174]^. In HCC, high expression of GLUT2 has been associated with poorer outcomes^[175,176]^. Furthermore, an elevated GLUT2 expression has also been found in more invasive versions of ductal carcinoma, tubular colon carcinoma, pancreatic adenocarcinoma, and pulmonary mesothelioma^[177]^. Whether *SLC2A2* genetic variants have an impact on the development of resistance to anticancer drugs and other malignant characteristics of these tumors is yet unknown.

### Amino acid transporters

The L-type amino acid transporter 1 (LAT1; *SLC7A5*)^[178]^, which preferentially transports large neutral amino acids, including most essential amino acids, is overexpressed in several types of cancer^[178,179]^. Some studies have suggested that LAT1 expression correlates with cancer cell growth and proliferation^[180]^, which has led to the proposal of LAT1 as a potential prognostic biomarker in different types of cancer^[181,182]^. Moreover, LAT1 is an appealing target for pharmacologically manipulating the uptake of cancer drugs and prodrugs, such as melphalan and acivicin^[183-185]^. Several compounds that specifically inhibit LAT1, like the novel tyrosine analog nanvuranlat (JPH203), have been developed to treat several types of solid tumors^[186-188]^. Genetic variants of *SLC7A5* have been associated with cancer pharmacology. Thus, the variant g.87889203G>A/C/T (rs4240803), both in homozygosis and heterozygosis, has been related to a decreased risk of melphalan-induced gastrointestinal toxicity in patients with multiple myeloma treated with this drug^[152]^ [Table 4].

### Folate transporters

Since folate is required for cancer cell proliferation, folate transporters play a relevant role in the response to antifolate drugs. Namely, two transporters must be considered: reduced folate transporter 1 (RFC1, *SLC19A1*), which is the primary transporter responsible for folate uptake widely expressed in the body^[189,190]^, and the proton-coupled folate transporter (PCFT, *SLC46A1*)^[191]^. RFC1 mediates high-affinity transport by tumor cells of antifolate chemotherapeutic agents, such as methotrexate and pemetrexed^[192,193]^, raltitrexed, and pralatrexate^[194,195]^. These drugs can also be transported, although to a lesser extent, by PCFT^[194,196,197]^.

High expression of RFC1 has been detected in NSCLC, squamous cell carcinoma, neuroblastoma, colorectal carcinoma, and urothelial bladder carcinomas^[198-200]^. Because antifolates are essential drugs in the treatment of numerous cancers, including pediatric ALL, osteogenic sarcoma, lymphoma, breast cancer, non-small cell lung cancer, and malignant pleural mesothelioma^[201-203]^, the appearance in tumor cells of *SLC19A1* variants associated with altered transport of these anticancer drugs, can result in poorer responses in these patients^[204]^. For instance, the SNP g.46957794T>C/G (rs1051266) has been associated with increased severity of adverse effects of methotrexate in patients with precursor cell lymphoblastic leukemia^[205]^. Moreover, the SNP g.46957794T>C/G (rs1051266), both in homozygosis and heterozygosis, has been associated with decreased plasma concentrations of methotrexate in children with osteosarcoma^[156]^. Finally, the SNP g.46948827G>A/C/T (rs4818789) is associated with a decreased risk of adverse effects when treated with cyclosporine and methotrexate in people with hemopoietic stem cell transplant^[206]^.

## CONCLUSION

Besides the complexity of the clinical problem posed by diverse and synergistic MOCs accounting for the lack of response of many cancers to pharmacological treatments, the available evidence supports the existence of a higher degree of complexity due to the appearance of genetic variants in the elements forming part of the resistome. The present review highlights the clinical impact of a subset of *MOC* genes belonging to the transportome, and more precisely, these transporters involved in drug uptake, which has been classified as MOC-1A subtype^[1,2]^. The presence of variants favoring the resistance acts as a Darwinian selection factor, thus contributing to developing a more malignant phenotype during cancer progression. Accordingly, the effort to achieve the desirable horizon of more personalized medicine to treat cancer patients requires the identification of this issue in all its dimensions, i.e., not only measuring the expression levels of every gene involved in the resistome but also characterizing the different variants that can affect their function. It is also essential to consider the time-dependent aspect of these features, as the genetic expression and the appearance of genetic variants can change during tumor progression and in response to treatment.

At present, evidence for the existence of an association between genetic variant-drug combinations is limited to genes encoding uptake transporters for antitumor drugs. Further research supporting such associations is needed to reach the level seen with more studied drugs, such as statins. For these drugs, a personalized genetic information influences the adjustment of their prescription, and clinical guidelines recommend testing each individual for genetic characteristics before drug administration.

## DECLARATIONS

### Copyright

© The Author(s) 2024.

## References

[1] Marin JJ, Monte MJ, Blazquez AG, Macias RI, Serrano MA, Briz O (2014). The role of reduced intracellular concentrations of active drugs in the lack of response to anticancer chemotherapy. Acta Pharmacol Sin.

[2] Marin JJG, Macias RIR, Monte MJ (2020). Molecular bases of drug resistance in hepatocellular carcinoma. Cancers.

[3] Marin JJG, Romero MR, Herraez E (2022). Mechanisms of pharmacoresistance in hepatocellular carcinoma: new drugs but old problems. Semin Liver Dis.

[4] Schaller L, Lauschke VM (2019). The genetic landscape of the human solute carrier (SLC) transporter superfamily. Hum Genet.

[5] Al-Abdulla R, Perez-Silva L, Abete L, Romero MR, Briz O, Marin JJG (2019). Unraveling “The Cancer Genome Atlas” information on the role of SLC transporters in anticancer drug uptake. Expert Rev Clin Pharmacol.

[6] Huang KM, Uddin ME, DiGiacomo D, Lustberg MB, Hu S, Sparreboom A (2020). Role of SLC transporters in toxicity induced by anticancer drugs. Expert Opin Drug Metab Toxicol.

[7] Baldwin SA, Beal PR, Yao SY, King AE, Cass CE, Young JD (2004). The equilibrative nucleoside transporter family, SLC29. Pflugers Arch.

[8] Mata JF, García-Manteiga JM, Lostao MP (2001). Role of the human concentrative nucleoside transporter (hCNT1) in the cytotoxic action of 5[Prime]-deoxy-5-fluorouridine, an active intermediate metabolite of capecitabine, a novel oral anticancer drug. Mol Pharmacol.

[9] Álvarez-Arenas A, Podolski-Renic A, Belmonte-Beitia J, Pesic M, Calvo GF (2019). Interplay of darwinian selection, lamarckian induction and microvesicle transfer on drug resistance in cancer. Sci Rep.

[10] Hagenbuch B, Stieger B (2013). The SLCO (former SLC21) superfamily of transporters. Mol Aspects Med.

[11] Hagenbuch B, Meier PJ (2004). Organic anion transporting polypeptides of the OATP/SLC21 family: phylogenetic classification as OATP/SLCO superfamily, new nomenclature and molecular/functional properties. Pflugers Arch.

[12] Schulte RR, Ho RH (2019). Organic anion transporting polypeptides: emerging roles in cancer pharmacology. Mol Pharmacol.

[13] Lee W, Glaeser H, Smith LH (2005). Polymorphisms in human organic anion-transporting polypeptide 1A2 (OATP1A2): implications for altered drug disposition and central nervous system drug entry. J Biol Chem.

[14] Badagnani I, Castro RA, Taylor TR (2006). Interaction of methotrexate with organic-anion transporting polypeptide 1A2 and its genetic variants. J Pharmacol Exp Ther.

[15] Hu S, Franke RM, Filipski KK (2008). Interaction of imatinib with human organic ion carriers. Clin Cancer Res.

[16] Yamakawa Y, Hamada A, Shuto T (2011). Pharmacokinetic impact of SLCO1A2 polymorphisms on imatinib disposition in patients with chronic myeloid leukemia. Clin Pharmacol Ther.

[17] Cheng Y, Chen MH, Zhuang Q (2021). Genetic factors involved in delayed methotrexate elimination in children with acute lymphoblastic leukemia. Pediatr Blood Cancer.

[18] Wang SM, Zeng WX, Wu WS, Sun LL, Yan D (2018). Association between a microRNA binding site polymorphism in SLCO1A2 and the risk of delayed methotrexate elimination in Chinese children with acute lymphoblastic leukemia. Leuk Res.

[19] Huang L, Zhang T, Xie C (2013). SLCO1B1 and SLC19A1 gene variants and irinotecan-induced rapid response and survival: a prospective multicenter pharmacogenetics study of metastatic colorectal cancer. PLoS One.

[20] Radtke S, Zolk O, Renner B (2013). Germline genetic variations in methotrexate candidate genes are associated with pharmacokinetics, toxicity, and outcome in childhood acute lymphoblastic leukemia. Blood.

[21] Bins S, Lenting A, El Bouazzaoui S (2016). Polymorphisms in SLCO1B1 and UGT1A1 are associated with sorafenib-induced toxicity. Pharmacogenomics.

[22] Reimer T, Kempert S, Gerber B, Thiesen HJ, Hartmann S, Koczan D (2016). SLCO1B1*5 polymorphism (rs4149056) is associated with chemotherapy-induced amenorrhea in premenopausal women with breast cancer: a prospective cohort study. BMC Cancer.

[23] Eldem İ, Yavuz D, Cumaoğullari Ö (2018). SLCO1B1 polymorphisms are associated with drug intolerance in childhood leukemia maintenance therapy. J Pediatr Hematol Oncol.

[24] Liu SG, Gao C, Zhang RD (2017). Polymorphisms in methotrexate transporters and their relationship to plasma methotrexate levels, toxicity of high-dose methotrexate, and outcome of pediatric acute lymphoblastic leukemia. Oncotarget.

[25] Han JY, Lim HS, Park YH, Lee SY, Lee JS (2009). Integrated pharmacogenetic prediction of irinotecan pharmacokinetics and toxicity in patients with advanced non-small cell lung cancer. Lung Cancer.

[26] Park HS, Lim SM, Shin HJ (2016). Pharmacogenetic analysis of advanced non-small-cell lung cancer patients treated with first-line paclitaxel and carboplatin chemotherapy. Pharmacogenet Genomics.

[27] Kloth JSL, Verboom MC, Swen JJ (2018). Genetic polymorphisms as predictive biomarker of survival in patients with gastrointestinal stromal tumors treated with sunitinib. Pharmacogenomics J.

[28] Chew SC, Sandanaraj E, Singh O (2012). Influence of SLCO1B3 haplotype-tag SNPs on docetaxel disposition in Chinese nasopharyngeal cancer patients. Br J Clin Pharmacol.

[29] Zhou F, Zheng J, Zhu L (2013). Functional analysis of novel polymorphisms in the human SLCO1A2 gene that encodes the transporter OATP1A2. AAPS J.

[30] (2018). Zamek-Gliszczynski MJ, Taub ME, Chothe PP, et al; International Transporter Consortium. Transporters in drug development: 2018 ITC recommendations for transporters of emerging clinical importance. Clin Pharmacol Ther.

[31] Kullak-Ublick GA, Ismair MG, Stieger B (2001). Organic anion-transporting polypeptide B (OATP-B) and its functional comparison with three other OATPs of human liver. Gastroenterology.

[32] Nagai M, Furihata T, Matsumoto S (2012). Identification of a new organic anion transporting polypeptide 1B3 mRNA isoform primarily expressed in human cancerous tissues and cells. Biochem Biophys Res Commun.

[33] Alam K, Farasyn T, Ding K, Yue W (2018). Characterization of liver- and cancer-type-organic anion transporting polypeptide (OATP) 1B3 messenger RNA expression in normal and cancerous human tissues. Drug Metab Lett.

[34] Al-Abdulla R, Perez-Silva L, Lozano E (2020). Sensitizing gastric adenocarcinoma to chemotherapy by pharmacological manipulation of drug transporters. Biochem Pharmacol.

[35] (2013). van de Steeg E, van Esch A, Wagenaar E, Kenworthy KE, Schinkel AH. Influence of human OATP1B1, OATP1B3, and OATP1A2 on the pharmacokinetics of methotrexate and paclitaxel in humanized transgenic mice. Clin Cancer Res.

[36] Asensio M, Herraez E, Macias RIR (2023). Relevance of the organic anion transporting polypeptide 1B3 (OATP1B3) in the personalized pharmacological treatment of hepatocellular carcinoma. Biochem Pharmacol.

[37] Garrison DA, Talebi Z, Eisenmann ED, Sparreboom A, Baker SD (2020). Role of OATP1B1 and OATP1B3 in drug-drug interactions mediated by tyrosine kinase inhibitors. Pharmaceutics.

[38] Zimmerman EI, Hu S, Roberts JL (2013). Contribution of OATP1B1 and OATP1B3 to the disposition of sorafenib and sorafenib-glucuronide. Clin Cancer Res.

[39] Ohya H, Shibayama Y, Ogura J, Narumi K, Kobayashi M, Iseki K (2015). Regorafenib is transported by the organic anion transporter 1B1 and the multidrug resistance protein 2. Biol Pharm Bull.

[40] Picard N, Levoir L, Lamoureux F, Yee SW, Giacomini KM, Marquet P (2011). Interaction of sirolimus and everolimus with hepatic and intestinal organic anion-transporting polypeptide transporters. Xenobiotica.

[41] Boivin AA, Cardinal H, Barama A (2013). Influence of SLCO1B3 genetic variations on tacrolimus pharmacokinetics in renal transplant recipients. Drug Metab Pharmacokinet.

[42] Gridelli C, Maione P, Rossi A (2008). The potential role of mTOR inhibitors in non-small cell lung cancer. Oncologist.

[43] Zagouri F, Sergentanis TN, Chrysikos D, Filipits M, Bartsch R (2012). mTOR inhibitors in breast cancer: a systematic review. Gynecol Oncol.

[44] Szklener K, Mazurek M, Wieteska M, Wacławska M, Bilski M, Mańdziuk S (2022). New directions in the therapy of glioblastoma. Cancers.

[45] Lee HH, Ho RH (2017). Interindividual and interethnic variability in drug disposition: polymorphisms in organic anion transporting polypeptide 1B1 (OATP1B1; SLCO1B1). Br J Clin Pharmacol.

[46] Crowe A, Zheng W, Miller J (2019). Characterization of plasma membrane localization and phosphorylation status of organic anion transporting polypeptide (OATP) 1B1 c.521 T>C nonsynonymous single-nucleotide polymorphism. Pharm Res.

[47] (2008). SEARCH Collaborative Group; Link E, Parish S, Armitage J, et al. SLCO1B1 variants and statin-induced myopathy - a genomewide study. N Engl J Med.

[48] Marin JJG, Serrano MA, Monte MJ (2020). Role of genetic variations in the hepatic handling of drugs. Int J Mol Sci.

[49] Ramsey LB, Gong L, Lee SB (2023). PharmVar GeneFocus: SLCO1B1. Clin Pharmacol Ther.

[50] Letschert K, Keppler D, König J (2004). Mutations in the SLCO1B3 gene affecting the substrate specificity of the hepatocellular uptake transporter OATP1B3 (OATP8). Pharmacogenetics.

[51] Lévi F, Karaboué A, Saffroy R (2017). Pharmacogenetic determinants of outcomes on triplet hepatic artery infusion and intravenous cetuximab for liver metastases from colorectal cancer (European trial OPTILIV, NCT00852228). Br J Cancer.

[52] Yu J, Zhou Z, Tay-Sontheimer J, Levy RH, Ragueneau-Majlessi I (2017). Intestinal drug interactions mediated by OATPs: a systematic review of preclinical and clinical findings. J Pharm Sci.

[53] Prasad B, Gaedigk A, Vrana M (2016). Ontogeny of hepatic drug transporters as quantified by LC-MS/MS proteomics. Clin Pharmacol Ther.

[54] Niemi M (2007). Role of OATP transporters in the disposition of drugs. Pharmacogenomics.

[55] Visentin M, Chang MH, Romero MF, Zhao R, Goldman ID (2012). Substrate- and pH-specific antifolate transport mediated by organic anion-transporting polypeptide 2B1 (OATP2B1-SLCO2B1). Mol Pharmacol.

[56] Fujita D, Saito Y, Nakanishi T, Tamai I (2016). Organic anion transporting polypeptide (OATP)2B1 contributes to gastrointestinal toxicity of anticancer drug SN-38, active metabolite of irinotecan hydrochloride. Drug Metab Dispos.

[57] Johnston RA, Rawling T, Chan T, Zhou F, Murray M (2014). Selective inhibition of human solute carrier transporters by multikinase inhibitors. Drug Metab Dispos.

[58] Medwid S, Price HR, Taylor DP (2021). Organic anion transporting polypeptide 2B1 (OATP2B1) genetic variants: in vitro functional characterization and association with circulating concentrations of endogenous substrates. Front Pharmacol.

[59] Lee HH, Leake BF, Teft W, Tirona RG, Kim RB, Ho RH (2015). Contribution of hepatic organic anion-transporting polypeptides to docetaxel uptake and clearance. Mol Cancer Ther.

[60] Buxhofer-Ausch V, Secky L, Wlcek K (2013). Tumor-specific expression of organic anion-transporting polypeptides: transporters as novel targets for cancer therapy. J Drug Deliv.

[61] Olszewski-Hamilton U, Svoboda M, Thalhammer T, Buxhofer-Ausch V, Geissler K, Hamilton G (2011). Organic anion transporting polypeptide 5A1 (OATP5A1) in small cell lung cancer (SCLC) cells: possible involvement in chemoresistance to satraplatin. Biomark Cancer.

[62] Nigam SK (2018). The SLC22 transporter family: a paradigm for the impact of drug transporters on metabolic pathways, signaling, and disease. Annu Rev Pharmacol Toxicol.

[63] Alexander SP, Kelly E, Mathie A (2021). The concise guide to pharmacology 2021/22: transporters. Br J Pharmacol.

[64] Lozano E, Briz O, Macias RIR, Serrano MA, Marin JJG, Herraez E (2018). Genetic heterogeneity of SLC22 family of transporters in drug disposition. J Pers Med.

[65] Zhu C, Nigam KB, Date RC (2015). Evolutionary analysis and classification of OATs, OCTs, OCTNs, and other SLC22 transporters: structure-function implications and analysis of sequence motifs. PLoS One.

[66] Koepsell H, Endou H (2004). The SLC22 drug transporter family. Pflugers Arch.

[67] Koepsell H (2015). Role of organic cation transporters in drug-drug interaction. Expert Opin Drug Metab Toxicol.

[68] Lozano E, Herraez E, Briz O (2013). Role of the plasma membrane transporter of organic cations OCT1 and its genetic variants in modern liver pharmacology. Biomed Res Int.

[69] Di Paolo A, Polillo M, Capecchi M (2014). The c.480C>G polymorphism of hOCT1 influences imatinib clearance in patients affected by chronic myeloid leukemia. Pharmacogenomics J.

[70] Qiu HB, Zhuang W, Wu T (2018). Imatinib-induced ophthalmological side-effects in GIST patients are associated with the variations of EGFR, SLC22A1, SLC22A5 and ABCB1. Pharmacogenomics J.

[71] Cargnin S, Ravegnini G, Soverini S, Angelini S, Terrazzino S (2018). Impact of SLC22A1 and CYP3A5 genotypes on imatinib response in chronic myeloid leukemia: A systematic review and meta-analysis. Pharmacol Res.

[72] Singh O, Chan JY, Lin K, Heng CC, Chowbay B (2012). SLC22A1-ABCB1 haplotype profiles predict imatinib pharmacokinetics in Asian patients with chronic myeloid leukemia. PLoS One.

[73] Ravegnini G, Urbini M, Simeon V (2019). An exploratory study by DMET array identifies a germline signature associated with imatinib response in gastrointestinal stromal tumor. Pharmacogenomics J.

[74] Angelini S, Soverini S, Ravegnini G (2013). Association between imatinib transporters and metabolizing enzymes genotype and response in newly diagnosed chronic myeloid leukemia patients receiving imatinib therapy. Haematologica.

[75] Angelini S, Pantaleo MA, Ravegnini G (2013). Polymorphisms in OCTN1 and OCTN2 transporters genes are associated with prolonged time to progression in unresectable gastrointestinal stromal tumours treated with imatinib therapy. Pharmacol Res.

[76] Seitz T, Stalmann R, Dalila N (2015). Global genetic analyses reveal strong inter-ethnic variability in the loss of activity of the organic cation transporter OCT1. Genome Med.

[77] Thomas J, Wang L, Clark RE, Pirmohamed M (2004). Active transport of imatinib into and out of cells: implications for drug resistance. Blood.

[78] Minematsu T, Giacomini KM (2011). Interactions of tyrosine kinase inhibitors with organic cation transporters and multidrug and toxic compound extrusion proteins. Mol Cancer Ther.

[79] Wang L, Giannoudis A, Lane S, Williamson P, Pirmohamed M, Clark R (2008). Expression of the uptake drug transporter hOCT1 is an important clinical determinant of the response to imatinib in chronic myeloid leukemia. Clin Pharmacol Ther.

[80] Nardinelli L, Sanabani SS, Didone A (2012). Pretherapeutic expression of the hOCT1 gene predicts a complete molecular response to imatinib mesylate in chronic-phase chronic myeloid leukemia. Acta Haematol.

[81] Makhtar SM, Husin A, Baba AA, Ankathil R (2018). Genetic variations in influx transporter gene *SLC22A1* are associated with clinical responses to imatinib mesylate among Malaysian chronic myeloid leukaemia patients. J Genet.

[82] Koren-Michowitz M, Buzaglo Z, Ribakovsky E (2014). OCT1 genetic variants are associated with long term outcomes in imatinib treated chronic myeloid leukemia patients. Eur J Haematol.

[83] Herraez E, Lozano E, Macias RI (2013). Expression of SLC22A1 variants may affect the response of hepatocellular carcinoma and cholangiocarcinoma to sorafenib. Hepatology.

[84] Geier A, Macias RI, Bettinger D (2017). The lack of the organic cation transporter OCT1 at the plasma membrane of tumor cells precludes a positive response to sorafenib in patients with hepatocellular carcinoma. Oncotarget.

[85] Zhang S, Lovejoy KS, Shima JE (2006). Organic cation transporters are determinants of oxaliplatin cytotoxicity. Cancer Res.

[86] Pérez-Gómez N, Fernández-Ortega MD, Elizari-Roncal M (2023). Identification of clinical and pharmacogenetic factors influencing metformin response in Type 2 diabetes mellitus. Pharmacogenomics.

[87] Koepsell H (2020). Organic cation transporters in health and disease. Pharmacol Rev.

[88] Fukushima-Uesaka H, Maekawa K, Ozawa S (2004). Fourteen novel single nucleotide polymorphisms in the SLC22A2 gene encoding human organic cation transporter (OCT2). Drug Metab Pharmacokinet.

[89] Visentin M, Torozi A, Gai Z (2018). Fluorocholine transport mediated by the organic cation transporter 2 (OCT2, SLC22A2): implication for imaging of kidney tumors. Drug Metab Dispos.

[90] Ciarimboli G, Holle SK, Vollenbröcker B (2011). New clues for nephrotoxicity induced by ifosfamide: preferential renal uptake via the human organic cation transporter 2. Mol Pharm.

[91] Nies AT, Schaeffeler E, Schwab M (2022). Hepatic solute carrier transporters and drug therapy: regulation of expression and impact of genetic variation. Pharmacol Ther.

[92] Chen L, Hong C, Chen EC (2013). Genetic and epigenetic regulation of the organic cation transporter 3, SLC22A3. Pharmacogenomics J.

[93] Gründemann D, Harlfinger S, Golz S (2005). Discovery of the ergothioneine transporter. Proc Natl Acad Sci U S A.

[94] Okabe M, Szakács G, Reimers MA (2008). Profiling SLCO and SLC22 genes in the NCI-60 cancer cell lines to identify drug uptake transporters. Mol Cancer Ther.

[95] Jong NN, Nakanishi T, Liu JJ, Tamai I, McKeage MJ (2011). Oxaliplatin transport mediated by organic cation/carnitine transporters OCTN1 and OCTN2 in overexpressing human embryonic kidney 293 cells and rat dorsal root ganglion neurons. J Pharmacol Exp Ther.

[96] Drenberg CD, Gibson AA, Pounds SB (2017). OCTN1 is a high-affinity carrier of nucleoside analogues. Cancer Res.

[97] Hu C, Lancaster CS, Zuo Z (2012). Inhibition of OCTN2-mediated transport of carnitine by etoposide. Mol Cancer Ther.

[98] Li Q, Shu Y (2014). Role of solute carriers in response to anticancer drugs. Mol Cell Ther.

[99] Koepsell H (2013). The SLC22 family with transporters of organic cations, anions and zwitterions. Mol Aspects Med.

[100] Nigam SK, Granados JC (2023). OAT, OATP, and MRP drug transporters and the remote sensing and signaling theory. Annu Rev Pharmacol Toxicol.

[101] Uwai Y, Taniguchi R, Motohashi H, Saito H, Okuda M, Inui K (2004). Methotrexate-loxoprofen interaction: involvement of human organic anion transporters hOAT1 and hOAT3. Drug Metab Pharmacokinet.

[102] Sun W, Wu RR, van Poelje PD, Erion MD (2001). Isolation of a family of organic anion transporters from human liver and kidney. Biochem Biophys Res Commun.

[103] Marada VV, Flörl S, Kühne A, Müller J, Burckhardt G, Hagos Y (2015). Interaction of human organic anion transporter 2 (OAT2) and sodium taurocholate cotransporting polypeptide (NTCP) with antineoplastic drugs. Pharmacol Res.

[104] Hagos Y, Wegner W, Kuehne A (2014). HNF4α induced chemosensitivity to oxaliplatin and 5-FU mediated by OCT1 and CNT3 in renal cell carcinoma. J Pharm Sci.

[105] Tashiro A, Tatsumi S, Takeda R (2014). High expression of organic anion transporter 2 and organic cation transporter 2 is an independent predictor of good outcomes in patients with metastatic colorectal cancer treated with FOLFOX-based chemotherapy. Am J Cancer Res.

[106] Shin HJ, Lee CH, Lee SS, Song IS, Shin JG (2010). Identification of genetic polymorphisms of human OAT1 and OAT2 genes and their relationship to hOAT2 expression in human liver. Clin Chim Acta.

[107] Cropp CD, Komori T, Shima JE (2008). Organic anion transporter 2 (SLC22A7) is a facilitative transporter of cGMP. Mol Pharmacol.

[108] Valdés R, Casado FJ, Pastor-Anglada M (2002). Cell-cycle-dependent regulation of CNT1, a concentrative nucleoside transporter involved in the uptake of cell-cycle-dependent nucleoside-derived anticancer drugs. Biochem Biophys Res Commun.

[109] Ritzel MW, Yao SY, Ng AM, Mackey JR, Cass CE, Young JD (1998). Molecular cloning, functional expression and chromosomal localization of a cDNA encoding a human Na+/nucleoside cotransporter (hCNT2) selective for purine nucleosides and uridine. Mol Membr Biol.

[110] Errasti-Murugarren E, Pastor-Anglada M, Casado FJ (2007). Role of CNT3 in the transepithelial flux of nucleosides and nucleoside-derived drugs. J Physiol.

[111] Kong W, Engel K, Wang J (2004). Mammalian nucleoside transporters. Curr Drug Metab.

[112] Pennycooke M, Chaudary N, Shuralyova I, Zhang Y, Coe IR (2001). Differential expression of human nucleoside transporters in normal and tumor tissue. Biochem Biophys Res Commun.

[113] Garcia-Manteiga J, Molina-Arcas M, Casado FJ, Mazo A, Pastor-Anglada M (2003). Nucleoside transporter profiles in human pancreatic cancer cells: role of hCNT1 in 2’,2’-difluorodeoxycytidine- induced cytotoxicity. Clin Cancer Res.

[114] Graham KA, Leithoff J, Coe IR (2000). Differential transport of cytosine-containing nucleosides by recombinant human concentrative nucleoside transporter protein hCNT1. Nucleosides Nucleotides Nucleic Acids.

[115] Jaramillo AC, Hubeek I, Broekhuizen R (2020). Expression of the nucleoside transporters hENT1 (SLC29) and hCNT1 (SLC28) in pediatric acute myeloid leukemia. Nucleosides Nucleotides Nucleic Acids.

[116] Niitani M, Nishida K, Okuda H, Nagai K, Fujimoto S, Nagasawa K (2010). Transport characteristics of mouse concentrative nucleoside transporter 1. Int J Pharm.

[117] Gray JH, Owen RP, Giacomini KM (2004). The concentrative nucleoside transporter family, SLC28. Pflugers Arch.

[118] Johnson SA (2001). Nucleoside analogues in the treatment of haematological malignancies. Expert Opin Pharmacother.

[119] Bhutia YD, Hung SW, Patel B, Lovin D, Govindarajan R (2011). CNT1 expression influences proliferation and chemosensitivity in drug-resistant pancreatic cancer cells. Cancer Res.

[120] Soo RA, Wang LZ, Ng SS (2009). Distribution of gemcitabine pathway genotypes in ethnic Asians and their association with outcome in non-small cell lung cancer patients. Lung Cancer.

[121] Innocenti F, Jiang C, Sibley AB (2019). An initial genetic analysis of gemcitabine-induced high-grade neutropenia in pancreatic cancer patients in CALGB 80303 (Alliance). Pharmacogenet Genomics.

[122] Khatri A, Williams BW, Fisher J (2014). SLC28A3 genotype and gemcitabine rate of infusion affect dFdCTP metabolite disposition in patients with solid tumours. Br J Cancer.

[123] Suenaga M, Schirripa M, Cao S (2017). Potential role of polymorphisms in the transporter genes ENT1 and MATE1/OCT2 in predicting TAS-102 efficacy and toxicity in patients with refractory metastatic colorectal cancer. Eur J Cancer.

[124] Tanaka M, Javle M, Dong X, Eng C, Abbruzzese JL, Li D (2010). Gemcitabine metabolic and transporter gene polymorphisms are associated with drug toxicity and efficacy in patients with locally advanced pancreatic cancer. Cancer.

[125] Lee SY, Im SA, Park YH (2014). Genetic polymorphisms of SLC28A3, SLC29A1 and RRM1 predict clinical outcome in patients with metastatic breast cancer receiving gemcitabine plus paclitaxel chemotherapy. Eur J Cancer.

[126] Limviphuvadh V, Tan CS, Konishi F (2018). Discovering novel SNPs that are correlated with patient outcome in a Singaporean cancer patient cohort treated with gemcitabine-based chemotherapy. BMC Cancer.

[127] Elsayed AH, Cao X, Crews KR (2018). Comprehensive Ara-C SNP score predicts leukemic cell intracellular ara-CTP levels in pediatric acute myeloid leukemia patients. Pharmacogenomics.

[128] Mitra AK, Kirstein MN, Khatri A (2012). Pathway-based pharmacogenomics of gemcitabine pharmacokinetics in patients with solid tumors. Pharmacogenomics.

[129] Shin HC, Landowski CP, Sun D (2003). Functional expression and characterization of a sodium-dependent nucleoside transporter hCNT2 cloned from human duodenum. Biochem Biophys Res Commun.

[130] Yee SW, Shima JE, Hesselson S (2009). Identification and characterization of proximal promoter polymorphisms in the human concentrative nucleoside transporter 2 (SLC28A2). J Pharmacol Exp Ther.

[131] Ritzel MWL, Ng AML, Yao SYM (2001). Molecular identification and characterization of novel human and mouse concentrative Na^+^-nucleoside cotransporter proteins (hCNT3 and mCNT3) broadly selective for purine and pyrimidine nucleosides (system *cib*). J Biol Chem.

[132] Song JH, Cho KM, Kim HJ (2015). Concentrative nucleoside transporter 3 as a prognostic indicator for favorable outcome of t(8;21)-positive acute myeloid leukemia patients after cytarabine-based chemotherapy. Oncol Rep.

[133] Fotoohi AK, Lindqvist M, Peterson C, Albertioni F (2006). Involvement of the concentrative nucleoside transporter 3 and equilibrative nucleoside transporter 2 in the resistance of T-lymphoblastic cell lines to thiopurines. Biochem Biophys Res Commun.

[134] Matimba A, Li F, Livshits A (2014). Thiopurine pharmacogenomics: association of SNPs with clinical response and functional validation of candidate genes. Pharmacogenomics.

[135] Jo JK, Oh JJ, Kim YT (2017). A genetic variant in SLC28A3, rs56350726, is associated with progression to castration-resistant prostate cancer in a Korean population with metastatic prostate cancer. Oncotarget.

[136] Clarke ML, Mackey JR, Baldwin SA, Young JD, Cass CE (2002). The role of membrane transporters in cellular resistance to anticancer nucleoside drugs. Cancer Treat Res.

[137] Reese ND, Schiller GJ (2013). High-dose cytarabine (HD araC) in the treatment of leukemias: a review. Curr Hematol Malig Rep.

[138] Kim KI, Huh IS, Kim IW (2013). Combined interaction of multi-locus genetic polymorphisms in cytarabine arabinoside metabolic pathway on clinical outcomes in adult acute myeloid leukaemia (AML) patients. Eur J Cancer.

[139] Amaki J, Onizuka M, Ohmachi K (2015). Single nucleotide polymorphisms of cytarabine metabolic genes influence clinical outcome in acute myeloid leukemia patients receiving high-dose cytarabine therapy. Int J Hematol.

[140] Wan H, Zhu J, Chen F (2014). SLC29A1 single nucleotide polymorphisms as independent prognostic predictors for survival of patients with acute myeloid leukemia: an in vitro study. J Exp Clin Cancer Res.

[141] Magos A, Baumann R, Turnbull AC (1988). Management of ruptured and unruptured ectopic pregnancies by videopelviscopy. Lancet.

[142] Petris MJ (2004). The SLC31 (Ctr) copper transporter family. Pflugers Arch.

[143] Ishida S, Lee J, Thiele DJ, Herskowitz I (2002). Uptake of the anticancer drug cisplatin mediated by the copper transporter Ctr1 in yeast and mammals. Proc Natl Acad Sci U S A.

[144] Holzer AK, Manorek GH, Howell SB (2006). Contribution of the major copper influx transporter CTR1 to the cellular accumulation of cisplatin, carboplatin, and oxaliplatin. Mol Pharmacol.

[145] Dasari S, Tchounwou PB (2014). Cisplatin in cancer therapy: molecular mechanisms of action. Eur J Pharmacol.

[146] Whirl-Carrillo M, McDonagh EM, Hebert JM (2012). Pharmacogenomics knowledge for personalized medicine. Clin Pharmacol Ther.

[147] Tate JG, Bamford S, Jubb HC (2019). COSMIC: the catalogue of somatic mutations in cancer. Nucleic Acids Res.

[148] Xu X, Duan L, Zhou B, Ma R, Zhou H, Liu Z (2012). Genetic polymorphism of copper transporter protein 1 is related to platinum resistance in Chinese non-small cell lung carcinoma patients. Clin Exp Pharmacol Physiol.

[149] Sun C, Zhang Z, Qie J (2018). Genetic polymorphism of *SLC31A1* is associated with clinical outcomes of platinum-based chemotherapy in non-small-cell lung cancer patients through modulating microRNA-mediated regulation. Oncotarget.

[150] Xu X, Ren H, Zhou B (2012). Prediction of copper transport protein 1 (CTR1) genotype on severe cisplatin induced toxicity in non-small cell lung cancer (NSCLC) patients. Lung Cancer.

[151] Drögemöller BI, Monzon JG, Bhavsar AP (2017). Association between SLC16A5 genetic variation and cisplatin-induced ototoxic effects in adult patients with testicular cancer. JAMA Oncol.

[152] Giglia JL, White MJ, Hart AJ (2014). A single nucleotide polymorphism in SLC7A5 is associated with gastrointestinal toxicity after high-dose melphalan and autologous stem cell transplantation for multiple myeloma. Biol Blood Marrow Transplant.

[153] Candelaria M, Ojeda J, Gutierrez-Hernandez O, Taja-Chayeb L, Vidal-Millan S, Duenas-Gonzalez A (2016). G80A single nucleotide polymorphism in reduced folate carrier-1 gene in a mexican population and its impact on survival in patients with acute lymphoblastic leukemia. Rev Invest Clin.

[154] Jabeen S, Holmboe L, Alnæs GI, Andersen AM, Hall KS, Kristensen VN (2015). Impact of genetic variants of RFC1, DHFR and MTHFR in osteosarcoma patients treated with high-dose methotrexate. Pharmacogenomics J.

[155] Suthandiram S, Gan GG, Zain SM (2014). Effect of polymorphisms within methotrexate pathway genes on methotrexate toxicity and plasma levels in adults with hematological malignancies. Pharmacogenomics.

[156] Park JA, Shin HY (2016). Influence of genetic polymorphisms in the folate pathway on toxicity after high-dose methotrexate treatment in pediatric osteosarcoma. Blood Res.

[157] Pongmaneratanakul S, Tanasanvimon S, Pengsuparp T, Areepium N (2017). Prevalence of CTR1 and ERCC1 polymorphisms and response of biliary tract cancer to gemcitabine-platinum chemotherapy. Asian Pac J Cancer Prev.

[158] Otsuka M, Matsumoto T, Morimoto R, Arioka S, Omote H, Moriyama Y (2005). A human transporter protein that mediates the final excretion step for toxic organic cations. Proc Natl Acad Sci U S A.

[159] Tanihara Y, Masuda S, Sato T, Katsura T, Ogawa O, Inui K (2007). Substrate specificity of MATE1 and MATE2-K, human multidrug and toxin extrusions/H^+^-organic cation antiporters. Biochem Pharmacol.

[160] Terada T, Inui K (2008). Physiological and pharmacokinetic roles of H^+^/organic cation antiporters (MATE/SLC47A). Biochem Pharmacol.

[161] Masuda S, Terada T, Yonezawa A (2006). Identification and functional characterization of a new human kidney-specific H^+^/organic cation antiporter, kidney-specific multidrug and toxin extrusion 2. J Am Soc Nephrol.

[162] Yonezawa A, Masuda S, Yokoo S, Katsura T, Inui K (2006). Cisplatin and oxaliplatin, but not carboplatin and nedaplatin, are substrates for human organic cation transporters (SLC22A1-3 and multidrug and toxin extrusion family). J Pharmacol Exp Ther.

[163] Yokoo S, Yonezawa A, Masuda S, Fukatsu A, Katsura T, Inui K (2007). Differential contribution of organic cation transporters, OCT2 and MATE1, in platinum agent-induced nephrotoxicity. Biochem Pharmacol.

[164] Nies AT, König J, Leuthold P (2023). Novel drug transporter substrates identification: An innovative approach based on metabolomic profiling, in silico ligand screening and biological validation. Pharmacol Res.

[165] Kajiwara M, Terada T, Ogasawara K (2009). Identification of multidrug and toxin extrusion (MATE1 and MATE2-K) variants with complete loss of transport activity. J Hum Genet.

[166] Teft WA, Winquist E, Nichols AC (2019). Predictors of cisplatin-induced ototoxicity and survival in chemoradiation treated head and neck cancer patients. Oral Oncol.

[167] Navale AM, Paranjape AN (2016). Glucose transporters: physiological and pathological roles. Biophys Rev.

[168] Pliszka M, Szablewski L (2021). Glucose transporters as a target for anticancer therapy. Cancers.

[169] Mano Y, Aishima S, Kubo Y (2014). Correlation between biological marker expression and fluorine-18 fluorodeoxyglucose uptake in hepatocellular carcinoma. Am J Clin Pathol.

[170] Yu M, Yongzhi H, Chen S (2017). The prognostic value of GLUT1 in cancers: a systematic review and meta-analysis. Oncotarget.

[171] Reinicke K, Sotomayor P, Cisterna P, Delgado C, Nualart F, Godoy A (2012). Cellular distribution of Glut-1 and Glut-5 in benign and malignant human prostate tissue. J Cell Biochem.

[172] Huang XQ, Chen X, Xie XX (2014). Co-expression of CD147 and GLUT-1 indicates radiation resistance and poor prognosis in cervical squamous cell carcinoma. Int J Clin Exp Pathol.

[173] Sobanski T, Rose M, Suraweera A, O’Byrne K, Richard DJ, Bolderson E (2021). Cell metabolism and DNA repair pathways: implications for cancer therapy. Front Cell Dev Biol.

[174] Chen J, Wu L, Wang Y (2016). Effect of transporter and DNA repair gene polymorphisms to lung cancer chemotherapy toxicity. Tumour Biol.

[175] Kim YH, Jeong DC, Pak K (2017). SLC2A2 (GLUT2) as a novel prognostic factor for hepatocellular carcinoma. Oncotarget.

[176] Daskalow K, Pfander D, Weichert W (2009). Distinct temporospatial expression patterns of glycolysis-related proteins in human hepatocellular carcinoma. Histochem Cell Biol.

[177] Godoy A, Ulloa V, Rodríguez F (2006). Differential subcellular distribution of glucose transporters GLUT1-6 and GLUT9 in human cancer: ultrastructural localization of GLUT1 and GLUT5 in breast tumor tissues. J Cell Physiol.

[178] Kanai Y, Segawa H, Miyamoto Ki, Uchino H, Takeda E, Endou H (1998). Expression cloning and characterization of a transporter for large neutral amino acids activated by the heavy chain of 4F2 antigen (CD98). J Biol Chem.

[179] Yanagida O, Kanai Y, Chairoungdua A (2001). Human L-type amino acid transporter 1 (LAT1): characterization of function and expression in tumor cell lines. Biochim Biophys Acta.

[180] Zhang J, Xu Y, Li D (2020). Review of the correlation of LAT1 with diseases: mechanism and treatment. Front Chem.

[181] Maimaiti M, Sakamoto S, Yamada Y (2020). Expression of L-type amino acid transporter 1 as a molecular target for prognostic and therapeutic indicators in bladder carcinoma. Sci Rep.

[182] Yanagisawa N, Ichinoe M, Mikami T (2012). High expression of L-type amino acid transporter 1 (LAT1) predicts poor prognosis in pancreatic ductal adenocarcinomas. J Clin Pathol.

[183] Lin J, Raoof DA, Thomas DG (2004). L-type amino acid transporter-1 overexpression and melphalan sensitivity in Barrett's adenocarcinoma. Neoplasia.

[184] Greig NH, Sweeney DJ, Rapoport SI (1987). Melphalan concentration dependent plasma protein binding in healthy humans and rats. Eur J Clin Pharmacol.

[185] Geier EG, Schlessinger A, Fan H (2013). Structure-based ligand discovery for the Large-neutral Amino Acid Transporter 1, LAT-1. Proc Natl Acad Sci U S A.

[186] Sekine M, Koh I, Nakamoto K (2023). Selective inhibition of L-type amino acid transporter 1 suppresses cell proliferation in ovarian clear cell carcinoma. Anticancer Res.

[187] Shi Z, Kaneda-Nakashima K, Ohgaki R (2023). Inhibition of cancer-type amino acid transporter LAT1 suppresses B16-F10 melanoma metastasis in mouse models. Sci Rep.

[188] Nishikubo K, Ohgaki R, Liu X (2023). Combination effects of amino acid transporter LAT1 inhibitor nanvuranlat and cytotoxic anticancer drug gemcitabine on pancreatic and biliary tract cancer cells. Cancer Cell Int.

[189] Sierra EE, Brigle KE, Spinella MJ, Goldman ID (1997). pH dependence of methotrexate transport by the reduced folate carrier and the folate receptor in L1210 leukemia cells. Further evidence for a third route mediated at low pH. Biochem Pharmacol.

[190] Puris E, Fricker G, Gynther M (2023). The role of solute carrier transporters in efficient anticancer drug delivery and therapy. Pharmaceutics.

[191] Qiu A, Jansen M, Sakaris A (2006). Identification of an intestinal folate transporter and the molecular basis for hereditary folate malabsorption. Cell.

[192] Alam C, Hoque MT, Finnell RH, Goldman ID, Bendayan R (2017). Regulation of reduced folate carrier (RFC) by vitamin D receptor at the blood-brain barrier. Mol Pharm.

[193] Westerhof GR, Schornagel JH, Kathmann I (1995). Carrier- and receptor-mediated transport of folate antagonists targeting folate-dependent enzymes: correlates of molecular-structure and biological activity. Mol Pharmacol.

[194] Desmoulin SK, Wang Y, Wu J (2010). Targeting the proton-coupled folate transporter for selective delivery of 6-substituted pyrrolo[2,3-d]pyrimidine antifolate inhibitors of de novo purine biosynthesis in the chemotherapy of solid tumors. Mol Pharmacol.

[195] Matherly LH, Hou Z, Deng Y (2007). Human reduced folate carrier: translation of basic biology to cancer etiology and therapy. Cancer Metastasis Rev.

[196] Lima A, Bernardes M, Azevedo R (2014). SLC19A1, SLC46A1 and SLCO1B1 polymorphisms as predictors of methotrexate-related toxicity in Portuguese rheumatoid arthritis patients. Toxicol Sci.

[197] Hou Z, Matherly LH Biology of the major facilitative folate transporters SLC19A1 and SLC46A1. Curr Top Membr.

[198] Lau DT, Flemming CL, Gherardi S (2015). MYCN amplification confers enhanced folate dependence and methotrexate sensitivity in neuroblastoma. Oncotarget.

[199] Odin E, Sondén A, Gustavsson B, Carlsson G, Wettergren Y (2015). Expression of folate pathway genes in stage III colorectal cancer correlates with recurrence status following adjuvant bolus 5-FU-based chemotherapy. Mol Med.

[200] Nunez MI, Behrens C, Woods DM (2012). High expression of folate receptor alpha in lung cancer correlates with adenocarcinoma histology and EGFR [corrected] mutation. J Thorac Oncol.

[201] Gonen N, Assaraf YG (2012). Antifolates in cancer therapy: structure, activity and mechanisms of drug resistance. Drug Resist Updat.

[202] Desmoulin SK, Hou Z, Gangjee A, Matherly LH (2012). The human proton-coupled folate transporter: Biology and therapeutic applications to cancer. Cancer Biol Ther.

[203] Visentin M, Zhao R, Goldman ID (2012). The antifolates. Hematol Oncol Clin North Am.

[204] Zhang X, Zhang D, Huang L (2019). Discovery of novel biomarkers of therapeutic responses in han chinese pemetrexed-based treated advanced NSCLC patients. Front Pharmacol.

[205] Salazar J, Altés A, del Río E (2012). Methotrexate consolidation treatment according to pharmacogenetics of MTHFR ameliorates event-free survival in childhood acute lymphoblastic leukaemia. Pharmacogenomics J.

[206] Laverdière I, Guillemette C, Tamouza R (2015). Cyclosporine and methotrexate-related pharmacogenomic predictors of acute graft-versus-host disease. Haematologica.

